# Computational insights into novel inhibitors: virtual screening of small molecules against human carbonic anhydrase II

**DOI:** 10.3389/fchem.2025.1627793

**Published:** 2025-10-02

**Authors:** Sermarajan Arunachalam, Balamurali M. M., Ramachandran Gnanasekaran

**Affiliations:** Department of Chemistry, School of Advanced Sciences, Vellore Institute of Technology, Chennai, India

**Keywords:** carbonic anhydrase, inhibitors, structure-based drug discovery, virtual screening, sulfonamide, molecular dynamics, pharmacophore modeling

## Abstract

Carbonic anhydrases, zinc-based metalloproteins, facilitate the reversible conversion of CO_2_ into carbonic acid when transported through blood vessels and subsequently regulate the physiological pH. In humans, this enzyme has been the therapeutic target for numerous diseases, as its abnormal regulation leads to a variety of disorders. The regulatory mechanism of this enzyme includes targeting catalytic Zn^2+^ ions as well as the residues that significantly regulate the protein’s structure and stability. With the available data on numerous sulfonamides, sulfamates, sulfamides, and non-sulfamide-derived inhibitors, in this study, a library of sulfonamide, extended aromatic sulfonamide, and non-sulfonamide derivatives was screened using a fragment-based drug discovery approach. Virtual screening was performed with molecular docking (DOCK 6 and Schrödinger GLIDE), rescored using MM-GBSA, and validated over 100-ns molecular dynamics simulations. Pharmacophore models were developed to identify key interaction features, while pharmacokinetic profiles were evaluated to assess their drug-likeness. Compounds S8 (sulfonamide) and S15–S16 (non-sulfonamides) emerged as promising inhibitors, showing strong Zn^2+^ coordination and stable binding to residues His93, Leu196, Thr197, and Thr198 that favor pharmacokinetic properties. The results provide atomistic insights into carbonic anhydrase II (CAII) inhibition and identify potential leads for further experimental validation.

## Graphical Abstract

**Figure:**
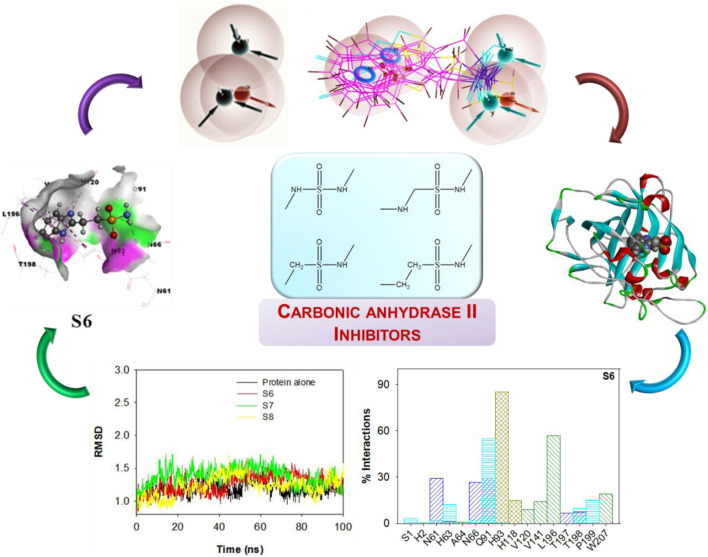

## Introduction

Metalloproteins with one or more metal ions are known to play crucial roles in numerous biological processes (Majorek et al., 2024). These proteins are significant as potential therapeutic targets as they serve crucial roles in catalyzing vital processes in various microbial metabolic pathways to regulate their pathogenicity (Capdevila et al., 2024; Murdoch and Skaar, 2022; Williams et al., 2023). Carbonic anhydrases (CAs) are one such zinc-based metalloprotein that has revealed its essential physiological roles in both prokaryotes and eukaryotes (Hirakawa et al., 2021). Moreover, CA is known to facilitate the conversion of CO_2_ into carbonic acid during its transportation by blood cells and subsequently reverse the process back to CO_2_. CA also plays a crucial role in regulating the physiological pH (Wang et al., 2025). In humans, 16 distinct drug-targeted isozymes of CA variants were reported, with each being responsible for unique physiological functions (Cuffaro et al., 2020; Imtaiyaz Hassan et al., 2013; Rai et al., 2022; Ronca and Supuran, 2024; Zamanova et al., 2019).

There are 15 isoforms of carbonic anhydrase (CAI to CAXV) in humans, with CAI–III, VII, and XIII being found in the cytoplasm, while CAIV, IX, XII, XIV, and XV are membrane bound, CAV is found in mitochondria, and CAVI is secreted in saliva. Many human CA variants have been proven as therapeutic targets for various illnesses, including diabetes, brain disorders, and, more recently, cancer (Garcia-Llorca et al., 2024; Yan et al., 2025; Zamanova et al., 2019). Among these isoforms, CAII has proven to be highly active and has been the target for numerous CA inhibitors. Moreover, CAII shares very high sequence similarity with CAI, CAIV, CAIX, and CAXII. Although the isomeric form CAII plays significant roles in metabolic pathways, its excess secretion may lead to a variety of disorders, including glaucoma, tubular kidney acidosis, and osteoporosis (Mishra and Sethi, 2025; Pastorekova et al., 2004).

Various mechanisms of inhibiting carbonic anhydrase were reported previously (Paciotti et al., 2025; Supuran, 2023). These include the binding of inhibitors to the catalytically essential Zn^2+^ ion in the enzyme’s active site, as well as various residues that are known to significantly regulate the protein’s structure and stability (Krishnamurthy et al., 2008; Thompson, 2022). Additionally, inhibitors containing phenols or carboxylates attach themselves to the zinc-coordinated hydrate or hydroxyl ion (Naeem et al., 2024). Recently, many novel CA inhibitors derived from sulfonamides, sulfamates, sulfamides, and non-sulfamide derivatives were reported (Al-Sanea et al., 2021; Bonardi et al., 2020; Pecina et al., 2018).

Zn^2+^ in the active site of CAII is known to make a strong coordinate bond with the incoming ligands. Recently, the associated energy transfer pathways upon the approach of the benzosulfonamide ligand toward the surrounding residues were evaluated (Gnanasekaran and Xu, 2022). It was revealed that small conformation changes (dihedral angle (H-N-S-C)) in the ligand inhibitors could affect the vibrational distribution within the molecule (Gnanasekaran and Pondy, 2024). These conformational variations could bring significant changes in the binding pocket of CAII. Considering the above results, herein, a series of ligands was identified that can effectively bind to Zn^2+^ and also interact with the surrounding neighboring atoms.

This investigation was carried out to identify novel inhibitors with strong binding affinities toward CA. CAII is frequently employed as a model system as its active site is highly conserved among the 15 human carbonic anhydrase isoforms. The catalytic zinc coordination sphere and key residues that mediate the binding of inhibitors are structurally similar across these isoforms. As a result, compounds that bind strongly to CAII often display comparable binding modes in other isoforms, making CAII an efficient and reliable alternative for screening and mechanistic studies of carbonic anhydrase inhibition.

A systematic study of the interactions of small molecular fragments could pave the way toward identifying high molecular mass drugs with enhanced potency. Therefore, the fragment-based drug discovery (FBDD) approach was adopted as a platform for identifying appropriate drug candidates in the process of drug discovery (Gamal et al., 2024; Mu et al., 2024; Pecina et al., 2018; Singh et al., 2025). Although numerous experimental studies have reported the interactions of large molecular inhibitors with the active site residues of CAII, the mechanistic details of how binding alters local interactions, energy transfer, and conformational dynamics remain poorly understood and are often left to speculation. Most prior reports focus on static binding affinities, without providing a molecular-level description of the pathways by which vibrational or electronic interactions propagate through the protein scaffold. Addressing this gap requires complementary computational approaches that can dissect both the binding energetics and the subsequent redistribution of energy within the enzyme–ligand complex (Gnansekaran and Xu, 2022).

In recent studies, we demonstrated how ligand binding modulates vibrational energy transfer pathways in CAII and how subtle conformational changes, such as dihedral angle variations, critically affect intramolecular energy distributions (Gnanasekaran and Pandey, 2024). Building on these findings, the present work provides new insights into CA inhibition mechanisms by systematically analyzing how small-molecule fragments—both sulfonamide and non-sulfonamide derivatives—interact not only with the catalytic Zn^2+^ but also with the surrounding residues that stabilize the active site. By combining structure-based molecular docking, MM-GBSA free energy calculations, pharmacophore profiling, and molecular dynamics simulations, we move beyond static binding descriptions and uncover the dynamic features of inhibitor-enzyme interactions. These results aim to bridge the existing gap between experimental observations and detailed atomistic mechanisms, thereby offering a more complete understanding of how CAII inhibition operates at the molecular level.

Herein, a series of organic molecules that include sulfonamide (-SO_2_-NH-) and non-sulfonamide moieties was identified from the ZINC database. Apart from the reported aromatic sulfonamide group (-SO_2_-NH-) compounds, this investigation included compounds that contain extended connectivity between the sulfonamide and the aromatic moiety through additional molecular linking moieties like -CH_2_-, -NH-, -CH_2_-CH_2_-, and -NH-CH_2_-*.* With the above library, a structure-based molecular docking was performed with CAII. Furthermore, MM-GBSA calculations were carried out to fetch more information on the binding affinity and free energy of the ligand–protein docked complexes. The selected compounds were assessed for maximum biocompatibility and drug likeness. The toxicity and pharmacophore activity of the compounds were predicted. Furthermore, the stability of interactions was analyzed through molecular dynamics simulations. These findings are believed to potentially provide inspiration for designing a CAII inhibitor.

## Methods

The crystal structure of CAII with a resolution of 1.65 Å and 260 residues was downloaded from the PDB databank (PDB Id: 3IGP) (Gitto et al., 2010). For the purpose of virtual screening, the inhibitors (non-sulfonamide organic molecules) were downloaded from the ZINC database (Tingle et al., 2023) with the criteria of molecular weight up to 200 Da, xlogP of 0.5, 1–3 rotatable bonds, 3 H-bond acceptors, 3 H-bond donors, and net charges varying from −2 to +2, while the other criteria in the database were maintained as is (Irwin et al., 2012). With the above criteria, 15,596 compounds were downloaded in SMILES format, and their three-dimensional structures were generated using Open Babel software (O'Boyle et al., 2011). The charges on the molecules were fixed using the ChimeraTool (Pettersen et al., 2004). The structures were docked using DOCK 6 software (Allen et al., 2015). Different energetically favorable poses were ranked based on the dock score and the distance (2.5 Å) between the Zn^2+^ and N atoms of the sulfonamide moiety. The hydrogen atoms of the selected best poses were again optimized employing the steepest descent algorithm in the CAII system to have the best “in vacuum” results using the Amber package (Case et al., 2005). They were again optimized using the SQM-based scoring function for which only the residues localized within a 10-Å distance from the active site were considered (Lepsik et al., 2013). Based on the binding energy, the top ∼186 ligands were selected. The geometries were visualized using BIOVIA Discovery Studio Viewer software 2021 (Biovia et al., 2016).

Schrödinger’s GLIDE was chosen for subsequent refinement and final pose evaluation because of its superior performance in pose prediction accuracy and scoring, as demonstrated in multiple comparative studies and reviews (Pagadala et al., 2017). Therefore, screening of the above ligands for their ability to bind effectively to the active site of CAII was carried out using the Schrödinger Maestro Suite 2020–23 software. The GLIDE module was employed to investigate the interactions and assess their binding affinities. Initially, the protein was preprocessed, optimized, and minimized under the protein preparation steps. The ligand was prepared using LigPrep part, which produced the maximum number of tautomers that helped to dock the ligand with different binding poses. The grid box was generated for both the ligands and CAII, and an accurate GLIDE score was obtained for the components present inside the grid box. Residues within a distance of 5 Å of the active site were considered. The selected ligand structures were then docked using Maestro, and score values were obtained for each ligand for only the energetically favorable different poses.

To these filtered structures, the MM-GBSA technique was used further to rescore and verify the docking results, with a flexible residue distance of 5 Å, and then assess the overall binding free energies. This also helped to eliminate false positives. The binding energies of the docked ligand–protein complexes were calculated using the formula

$$∆G=∆G_{\text{Bind}}\text{ coulomb}+∆G_{\text{Bind}}\text{ covalent}+∆G_{\text{Bind}}\;H‐\text{Bond}+∆G_{\text{Bind}}\text{ lipo}+∆G_{\text{Bind}}\text{ packing}+∆G_{\text{Bind}}\text{ self cont}+∆G_{\text{Bind}}\text{ Solv GB}+∆G_{\text{Bind}}\text{ vdW}.$$

Here, ∆G is the change in free energy of protein–ligand docked complexes. The parameters such as the change in binding free energy for Coulombic, covalent, hydrogen bonding, lipophilic (hydrophobic), packing, self-contact, solvation, and π–π stacking interactions are accounted together for the change in free energy of protein–ligand docked complexes. The binding free energy of packaging has contributions from several other parameters, including the contact and steric interaction between ligand and receptor, van der Waals forces, lipophilic interactions, and reduced solvent exposure (buried non-polar surfaces) (Friesner et al., 2004).

Molecular dynamics simulation (100 ns) was carried out using the DESMON functionality in Schrödinger Maestro software. In the system builder panel, the docked protein–ligand complexes were placed within an orthorhombic box using the TIP3P solvent model. To attain a physiological concentration of 0.15 M of sodium chloride (NaCl), counter ions were introduced to neutralize the solvated system. The NPT ensemble was used in the simulations, with the OPLS4 force field set for 100 ns at 300 K and 1.013 bar of atmospheric pressure (Gamal et al., 2024; Mu et al., 2024; Pecina et al., 2018).

Much of the costly drug development process has been a failure in the latter stage of clinical trials. Hence, computational assessment of absorption, distribution, metabolism, excretion, and toxicity (ADMET) properties helps to screen out non-drug materials and prevent mistaken identification, thereby addressing these financial challenges. The ADMET properties of the ligands were required for identifying prospective drugs. AI progress has facilitated the systematic evaluation of ADMET characteristics. Pharmacokinetic studies of absorption, distribution, metabolism, excretion, and toxicity were conducted to identify and eliminate false positives in the drug discovery process. The QikProp tool from the Schrödinger Suite was used to forecast the ADMET parameters for the suggested ZINC database ligands. Furthermore, the physicochemical and pharmacokinetic characteristics were evaluated to determine possible drug-likeness. The toxicity level of the drug was determined using the online browser ProTox-II.

## Results and discussion

**FIGURE 1:**
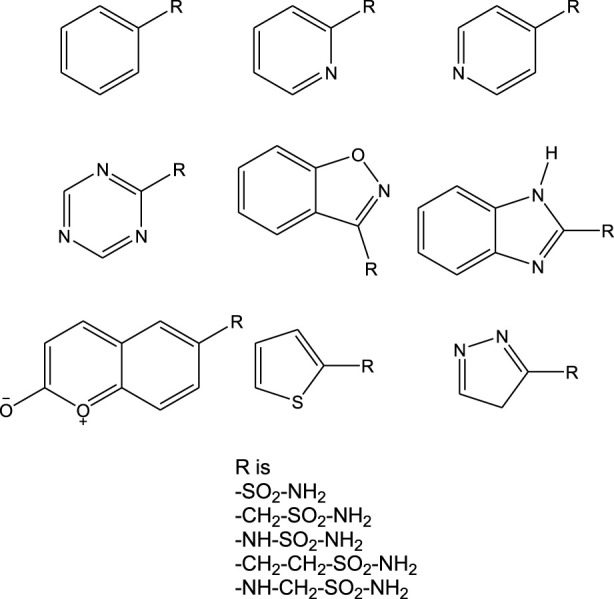
Chemical structures of various sulfonamide derivatives.

Analysis of the interactions of various small-molecule ligands with the Zn^2+^ of CAII revealed that the stability of the protein–ligand complexes was greatly influenced by the nature of functional groups, more specifically, the sulfonamide group. Non-sulfonamide ligands have also shown good affinity for CAII but were not considered for several reasons (Bozdag et al., 2019). The structures of a few sulfonamide derivatives considered for this investigation are shown in Figure 1.

### Preliminary screening: generation of pharmacophore hypothesis for various sulfonamide and non-sulfonamide ligands

Literature reports on large molecule inhibitors, particularly with sulfonamide functional moieties, wherein the sulfonamide group is directly linked to 5- or 6-membered aromatic ring systems, are available (Pecina et al., 2018; Singh et al., 2025). However, non-sulfonamide inhibitors are rarely reported because a weaker interaction is exhibited with the binding pocket residues, along with significant toxicity values (Cheng et al., 2024; Pontecorvi et al., 2025). These non-sulfonamide inhibitors were found to possess chemical moieties like PO_3_^−^, COS^−^, and COO^−^ (data not reported). Currently, investigations on sulfonamide-based inhibitors are being prioritized, exploring the possible gaps that can enhance the activity of the existing inhibitors. Several experimental reports offer outstanding inhibition potentials but lack mechanistic details. Herein, efforts were taken to investigate the introduction of linkers with different lengths between the sulfonamide moiety and aromatic rings. Moreover, literature reports have revealed that sulfonamide-based inhibitors exhibit stronger interactions and better inhibition potential than non-sulfonamide derivatives. Hence, this study might emphasize the key atomistic interactions that can influence the inhibition potential of sulfonamide derivatives effectively.

To narrow the library of inhibitor ligand molecules, pharmacophoric analysis was carried out on a relative scale through the assignment of positives and negatives.

**FIGURE 2:**
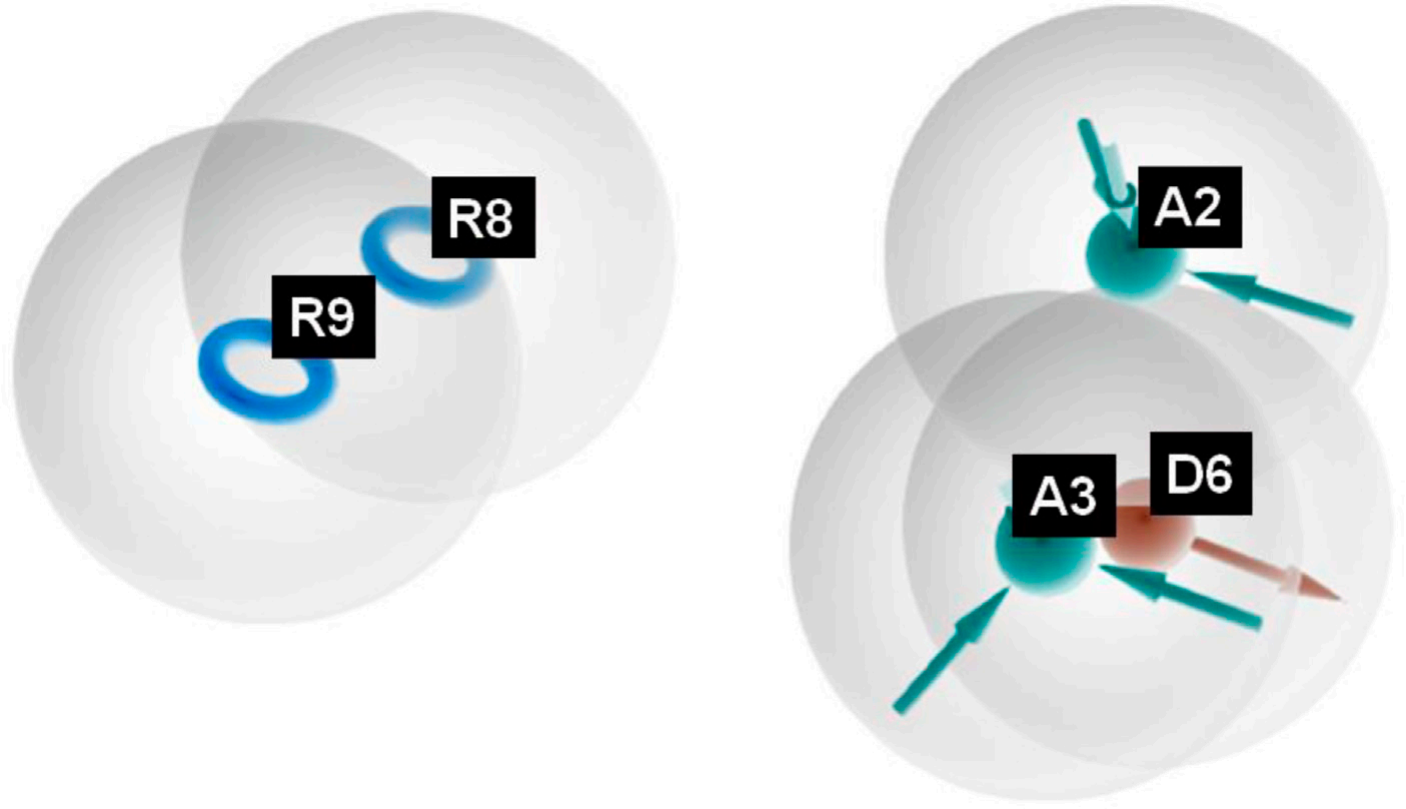
Pharmacophoric features obtained from the generated sulfonamide-derived ligand models (R, ionizable and aromatic ring; A, hydrogen bond acceptor; D, hydrogen bond donor).

**FIGURE 3:**
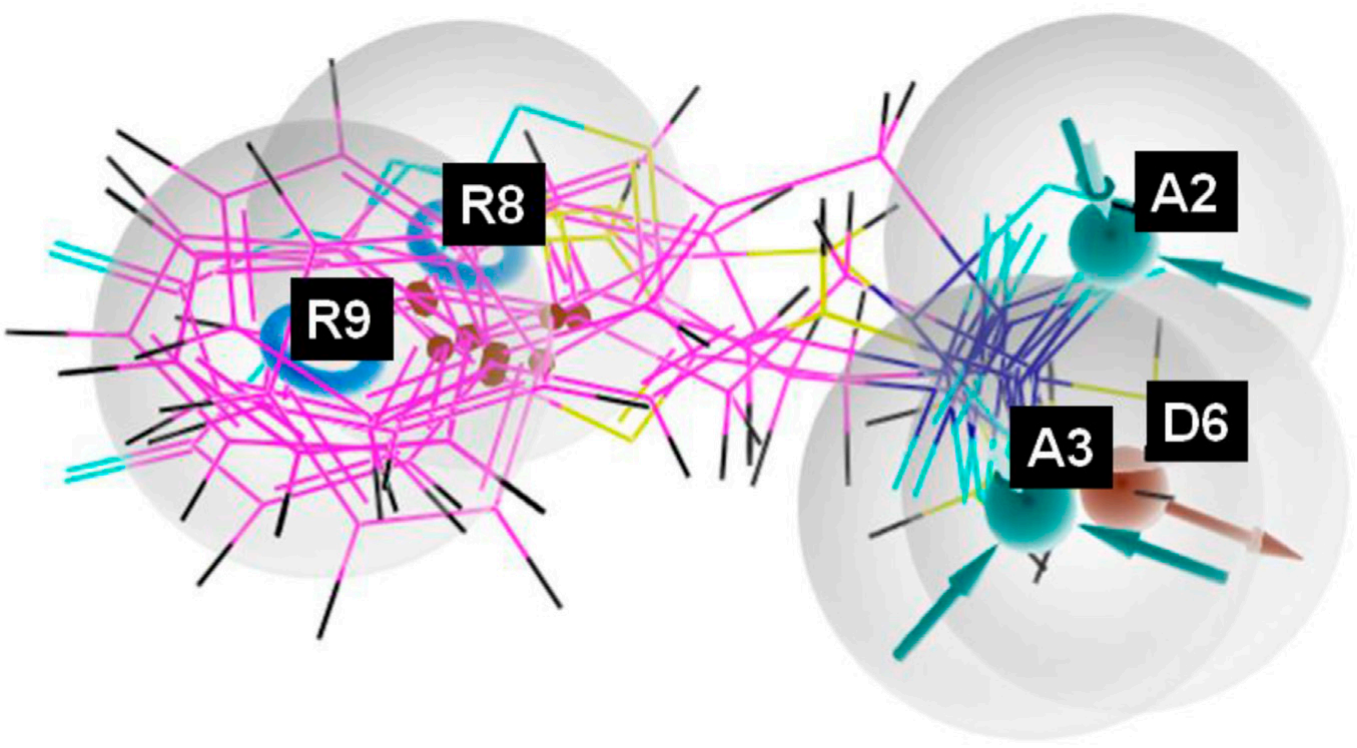
Pharmacophore model depicting the overlaid alignment of various active sulfonamide-derived ligands along the predicted features in the dataset.

The pharmacophore hypothesis for various CAII inhibitors was constructed using the phase module (Schrödinger suite 2023–2024). From the values of dock score and binding free energies, thresholds were assigned as the highest and lowest affinity scores. Non-covalent interactions with receptor sites could be simplified by positioning the pharmacophoric regions within a defined three-dimensional space. The pharmacophoric sites were constructed by utilizing the hydrogen bond donor (D), hydrogen bond acceptor (A), hydrophobic group (H), and positive (P) and negative (N) ionizable and aromatic ring (R), as described in the module phase. Of 186 ligands, 56 (32 sulfonamides and 24 non-sulfonamides) were selected as training sets by their wide range of activity and diversity, as predicted by the observed best and poor docking scores. The other 130 compounds served as test sets to facilitate a meaningful comparison with the predicted models. The pharmacophore hypotheses were assessed using their scoring function to maximize the alignment of ligands with high affinity while also including characteristics from ligands with low affinity to enhance the model’s versatility. The pharmacophore hypothesis was produced using 29 active compounds, with 1Å denoting the pharmacophore-matching tolerances among two pharmacophoric characteristics and 2Å representing the lowest inter-site distance. Ten different hypotheses were developed, with two serving as the maximum number of sites, with 50% serving as actives. The best of the ten produced hypotheses was chosen based on the number of matches, volume, vector, energy terms, site score, survival inactive, and survival score (Table 1). Different pharmacophore models (A2, A3, D6, R8, and R9) are shown in Figure 2, and the hypotheses scores are given in Table 1. The best survival score (AADRR_1) indicates a better alignment of the active ligand with the target site and will be a potent pharmacophore model. In the generated pharmacophore hypothesis, the ligands with higher survival scores are known to exhibit closer geometric alignment with the model’s essential features, indicating their fit to the binding template is with minimal distortion. Similarly, a higher vector score signifies that directional interactions like hydrogen bonds or metal coordinations are oriented optimally toward the receptor site. Together, these metrics will help to identify compounds whose chemical features are correctly positioned and pointed to engage the protein efficiently. The alignment of various sulfonamide inhibitors along with the various pharmacophoric features is depicted in Figure 3 (also in Supplementary Figure S1) and associated scores for the best hypothesis are given in Table 2 (see Supplementary Table S3 for the parametric scores of AADRR_3).

**TABLE 1:** Generated pharmacophore hypotheses for various sulfonamide-derived ligands based on their affinity toward the active site of CAII.

HypoID	Fitness	Survival	Site	Vector	Volume	Select	Matches	Inactive	Adjusted
AADRR_1	1.562	4.7715	0.692	0.9108	0.8432	1.3713	9	1.8553	2.9162
AADRR_2	1.500	4.7016	0.6761	0.8879	0.8181	1.3653	9	1.8529	2.8486
AADRR_3	1.420	4.6915	0.5864	0.9283	0.8216	1.4010	9	1.7657	2.9258
AADRR_4	1.433	4.6662	0.5944	0.8938	0.8294	1.3943	9	1.6994	2.9668
AADRR_5	1.508	4.6588	0.6354	0.9113	0.7970	1.3608	9	1.7976	2.8612
AADRR_6	1.587	4.6336	0.5680	0.9145	0.7990	1.3978	9	1.8342	2.7994
AADRR_7	1.429	4.6013	0.5444	0.9078	0.7982	1.3967	9	1.7033	2.8980
AADRR_8	1.353	4.5632	0.5129	0.9137	0.7907	1.3917	9	1.7156	2.8476
AADRR_9	1.769	4.5405	0.5118	0.9332	0.7693	1.3720	9	2.0488	2.4917
AADRR_10	1.703	4.5188	0.5790	0.8527	0.7694	1.3634	9	1.9267	2.5921

**TABLE 2:** Different parametric scores of the generated hypothesis AADRR_8.

Hypothesis	Activity	Fitness	Site score	Vector score	Volume	Matched ligand site	
AADRR_8	Active	1.599	0.178	0.8	0.621	A(2) A(3) D(5) R(7) R(8)	S11
	Active	1.804	0.216	0.869	0.718	A(4) A(2) D(7) R(10) R(11)	S161
	Active	2.651	0.78	0.995	0.876	A(3) A(2) D(6) R(7) R(8)	S152
	Active	3	1	1	1	A(2) A(3) D(6) R(8) R(9)	S169
	Active	2.999	0.999	1	1	A(2) A(3) D(5) R(8) R(9)	S169
	Active	2.991	0.993	1	0.998	A(2) A(3) D(6) R(8) R(9)	S169
	Active	1.772	0.265	0.784	0.723	A(2) A(3) D(5) R(7) R(8)	S158
	Active	2.11	0.357	0.987	0.766	A(2) A(1) D(6) R(8) R(7)	S157
	Active	1.814	0.315	0.875	0.624	A(1) A(2) D(5) R(8) R(7)	S150
	Inactive	1.775	0.305	0.986	0.555	A(3) A(2) D(4) R(6) R(−)	S181
	Inactive	1.355	0.275	0.627	0.515	A(2) A(3) D(5) R(7) R(−)	S175
	Inactive	1.786	0.341	0.968	0.558	A(2) A(3) D(4) R(7) R(−)	S175
	Inactive	1.906	0.457	0.977	0.59	A(2) A(3) D(5) R(7) R(−)	S175
	Inactive	1.769	0.3	0.986	0.552	A(4) A(3) D(6) R(8) R(−)	S178
	Inactive	1.742	0.348	0.942	0.534	A(3) A(4) D(5) R(8) R(−)	S170
	Inactive	1.96	1	1	0.592	A(3) A(2) D(4) R(−) R(−)	S166
	Inactive	2.026	1	1	0.659	A(3) A(2) D(5) R(−) R(−)	S156
	Inactive	1.862	0.529	0.822	0.654	A(1) A(2) D(6) R(−) R(8)	S149
	Inactive	1.777	1	1	0.41	A(4) A(3) D(5) R(−) R(−)	S174
	Inactive	1.773	0.997	1	0.406	A(4) A(3) D(5) R(−) R(−)	S174
	Inactive	1.469	0.171	0.857	0.479	A(4) A(3) D(6) R(7) R(−)	S172
	Inactive	1.355	0.152	0.754	0.482	A(4) A(3) D(5) R(8) R(−)	S184
	Inactive	1.353	0.148	0.755	0.482	A(4) A(3) D(5) R(8) R(−)	S184
	Inactive	1.824	0.649	0.8	0.57	A(3) A(4) D(8) R(10) R(−)	S8

**FIGURE 4:**
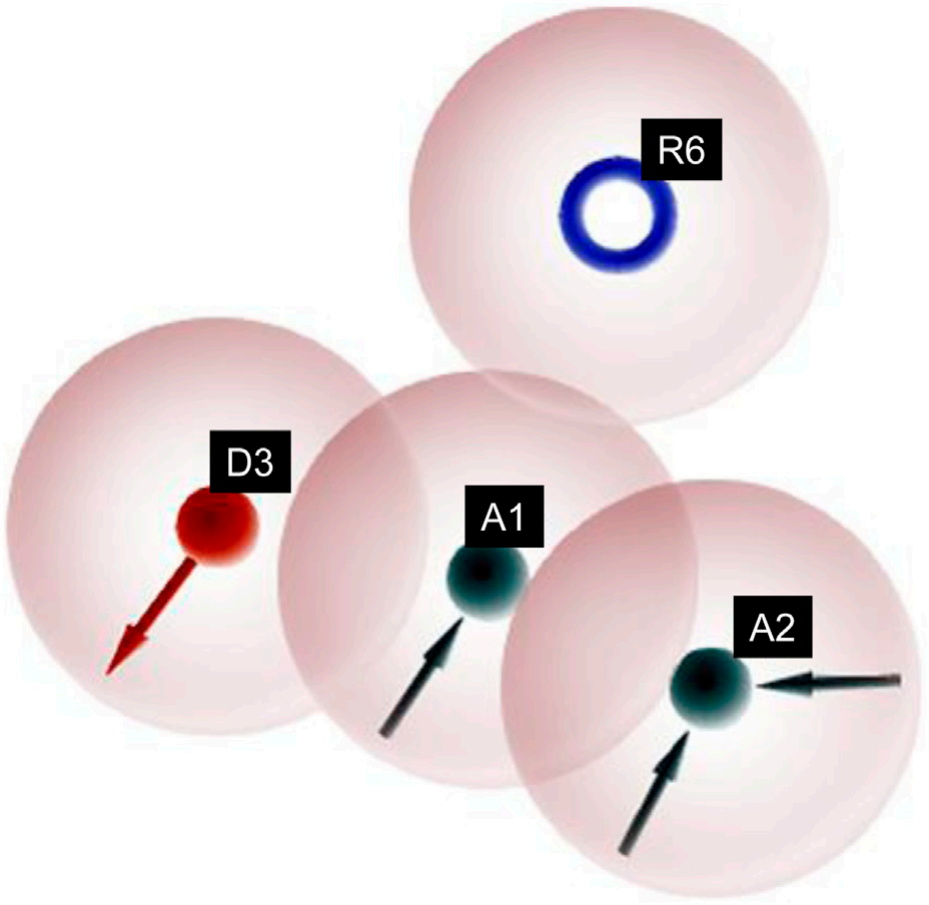
Pharmacophoric features obtained from the generated non-sulfonamide-derived ligand models.

**FIGURE 5:**
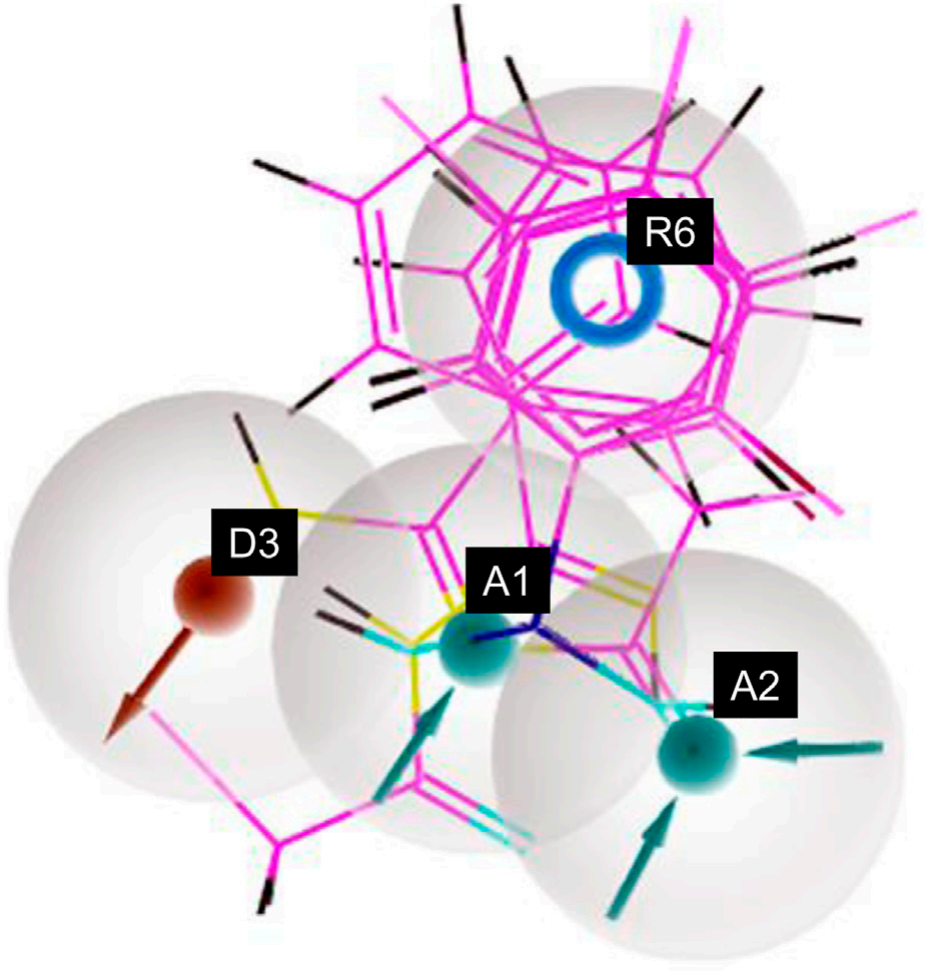
Pharmacophore model depicting the features predicted for various non-sulfonamide-derived ligands in the dataset.

Similarly, different pharmacophore models and their correspondingly derived hypotheses are shown in Figure 4, and associated features are given in Table 3. The alignments of various non-sulfonamide inhibitors along with the various pharmacophoric scores are depicted in Figure 4 (also in Supplementary Figure S2) and Table 3. The associated scores for the best hypothesis (AADR_1) are given in Table 4 (also see Supplementary Table S4 for the parametric scores of AADH_1). The alignments of various non-sulfonamide inhibitors along with the various pharmacophoric features are depicted in Figure 5.

**TABLE 3:** Generated pharmacophore hypotheses for various non-sulfonamide-derived ligands based on their affinity toward the active site of CAII enzyme.

HypoID	Fitness	Survival	Site	Vector	Volume	Select	Matches	Inactive	Adjusted
AADHR_1	0.864	4.2011	0.4407	0.7565	0.7274	1.4984	6	1.5223	2.6788
AADHR_2	1.007	4.1925	0.4639	0.7460	0.7039	1.5005	6	1.5501	2.6424
AADHR_3	1.007	4.1848	0.4705	0.7360	0.6996	1.5005	6	1.5391	2.6457
DDPR_1	1.709	5.7781	0.9987	1.0000	0.9406	1.9938	7	1.8251	3.9530
DDPR_2	1.248	5.5887	0.8509	0.9547	0.8819	1.9981	8	1.7428	3.8460
AAHR_1	1.644	4.2470	0.6237	0.8791	0.6992	1.2000	7	1.8632	2.3838
ADPR_1	1.311	4.1002	0.3067	0.5924	0.6249	1.6732	8	1.7886	2.3116
ADHR_1	0.907	4.0325	0.486	0.8350	0.6676	1.2657	6	1.6842	2.3483
ADHR_2	1.220	3.9892	0.5041	0.6971	0.7070	1.3028	6	1.6685	2.3208
ADHR_3	1.213	3.9798	0.5164	0.6812	0.7012	1.3028	6	1.6273	2.3524
ADHR_4	1.229	3.9668	0.4453	0.7099	0.7364	1.2971	6	1.7068	2.2600
AADR_1	0.534	3.6199	0.3294	0.6911	0.6817	0.9635	9	1.4407	2.1792
AADH_1	0.715	3.4655	0.3319	0.6716	0.6185	0.9984	7	1.5770	1.8884

**TABLE 4:** Different parametric scores of the generated hypothesis AADR_1.

Hypothesis	Activity	Fitness	Site score	Vector score	Volume	Matched ligand site	
AADR_1	Active	1.884	0.412	0.749	0.723	A(2) A(1) D(4) R(7)	S3
	Active	1.871	0.413	0.749	0.709	A(1) A(2) D(3) R(8)	S4
	Active	1.878	0.412	0.759	0.706	A(1) A(2) D(3) R(8)	S7
	Active	1.874	0.413	0.749	0.713	A(2) A(1) D(4) R(8)	S11
	Active	1.851	0.412	0.759	0.679	A(2) A(1) D(3) R(7)	S13
	Active	1.851	0.412	0.759	0.679	A(1) A(2) D(3) R(7)	S14
	Active	1.876	0.412	0.749	0.715	A(2) A(1) D(4) R(8)	S17
	Active	3.000	1.000	1.000	1.000	A(1) A(2) D(3) R(6)	S100
	Active	0.534	−0.252	0.257	0.529	A(1) A(2) D(3) R(6)	S100
	Inactive	1.836	0.647	0.852	0.569	A(2) A(1) D(−) R(5)	S48
	Inactive	2.013	0.886	0.833	0.690	A(2) A(1) D(−) R(6)	S105
	Inactive	1.388	0.428	0.653	0.439	A(1) A(2) D(4) R(−)	S107
	Inactive	1.140	0.388	0.458	0.411	A(1) A(2) D(4) R(−)	S122
	Inactive	1.300	0.412	0.648	0.366	A(2) A(3) D(4) R(−)	S123
	Inactive	1.360	0.408	0.677	0.399	A(2) A(3) D(4) R(−)	S124
	Inactive	1.163	0.355	0.572	0.340	A(2) A(1) D(4) R(−)	S129
	Inactive	1.174	0.355	0.572	0.352	A(2) A(1) D(4) R(−)	S130
	Inactive	1.329	0.435	0.654	0.374	A(1) A(3) D(4) R(−)	S132
	Inactive	1.273	0.428	0.630	0.347	A(1) A(3) D(4) R(−)	S132
	Inactive	1.321	0.910	0.601	0.226	A(3) A(2) D(6) R(−)	S133
	Inactive	1.306	0.428	0.63	0.381	A(1) A(3) D(4) R(−)	S133
	Inactive	1.166	0.316	0.396	0.546	A(2) A(1) D(4) R(−)	S134
	Inactive	1.033	0.36	0.55	0.229	A(1) A(2) D(5) R(−)	S141
	Inactive	1.592	0.249	0.751	0.593	A(1) A(2) D(4) R(5)	S79
	Inactive	1.575	0.249	0.751	0.576	A(1) A(2) D(4) R(6)	S81
	Inactive	1.776	0.264	0.84	0.672	A(3) A(2) D(6) R(8)	S87
	Inactive	1.712	0.289	0.71	0.713	A(3) A(2) D(5) R(8)	S87
	Inactive	1.714	0.291	0.71	0.714	A(2) A(1) D(4) R(8)	S87
	Inactive	1.643	0.278	0.749	0.615	A(1) A(2) D(4) R(6)	S96

**FIGURE 6:**
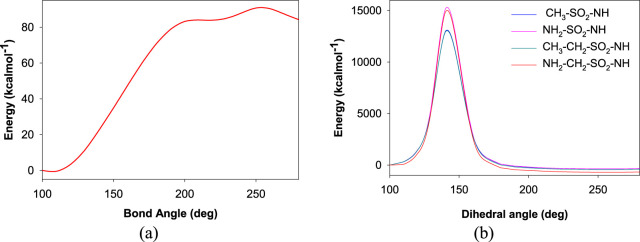
Plot depicting the potential energy surface for variations in the **(a)** bond angle and **(b)** dihedral angle between the sulfonamide moiety and the rest of the aromatic ring.

**FIGURE 7:**
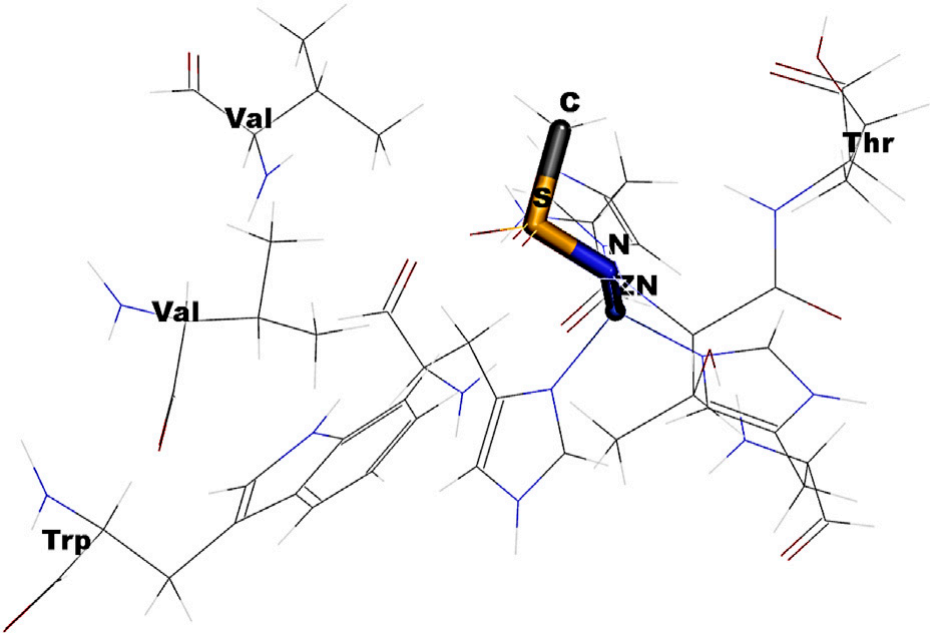
Schematic depicting the steric hindrance induced by the change in the dihedral angle rotation of HN-S-NH.

Further investigations were carried out with the identified ligand structures, focusing on the functional group fragment. The three-dimensional structures of ligands were generated using Open Babel software (Pandey and Pandey, 2024), with further modifications considering recent synthetic advances in heteroaryl functionalization [Ref]. Organic Letters, 27(22), 5625-5631. 10.1021/acs.orglett.5c01350. Herein, the influence of additional linking moieties like CH_2_-, -NH-, -CH_2_-CH_2_-, and -NH-CH_2_- on the binding interactions with the active site of CAII was investigated. As a proof of concept, an initial rigid potential energy surface (PES) scan was performed for the interaction of representative ligands with the residues localized within 5 Å from Zn^2+^ of CAII by varying the bond angle and dihedral angle using the B3LYP/6-31G method (Figure 6). The observation of a barrier along the surface indicates that the structural features of the ligands significantly influence their interaction with the active site residues, along with Zn^2+^. Thus, the observed variations in energy along the potential energy surface revealed that tuning the nature of functional groups on the ligands could pave the way to discover novel inhibitors. The induction of steric hindrance on the HN-S-NH moiety due to a change in the dihedral angle is shown in Figure 7.

**FIGURE 8:**
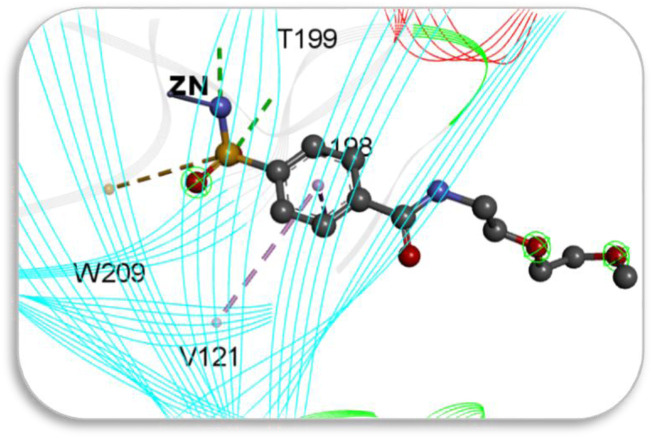
Docked pose of a drug interacting with the residues in the cavity of 2FOS.

In Figure 6A, the observed barrier (∼3.9 eV) could be due to the increase in the steric factor as the bond angle increases. The binding cavity void may not be large enough for any significant fluctuations of the interacting functional groups. The same is true for the variation in dihedral angle (Figure 6B). In the latter case, it was observed that at particular orientations of the functional groups with respect the aromatic skeleton, the strain energy has increased, which could be the possible reason for the observed barriers of 655.60 eV, 642.36 eV, 655.60 eV, and 559.09 eV, respectively, for the -NH-SO_2_-NH-, -NH-CH_2_-SO_2_-NH-, -CH_2_-SO_2_-NH-, and -CH_2_-CH_2_-SO_2_-NH- moieties along the PES. The reported binding pose of the benzenesulfonamide prong in the active site of human carbonic anhydrases I and II is shown in Figure 8. It can be seen that the amine group of the benzenesulfonamide ligand is coordinated to the active site Zn^2+^ (Elsayad et al., 2025).

### Molecular screening using GLIDE

**FIGURE 9:**
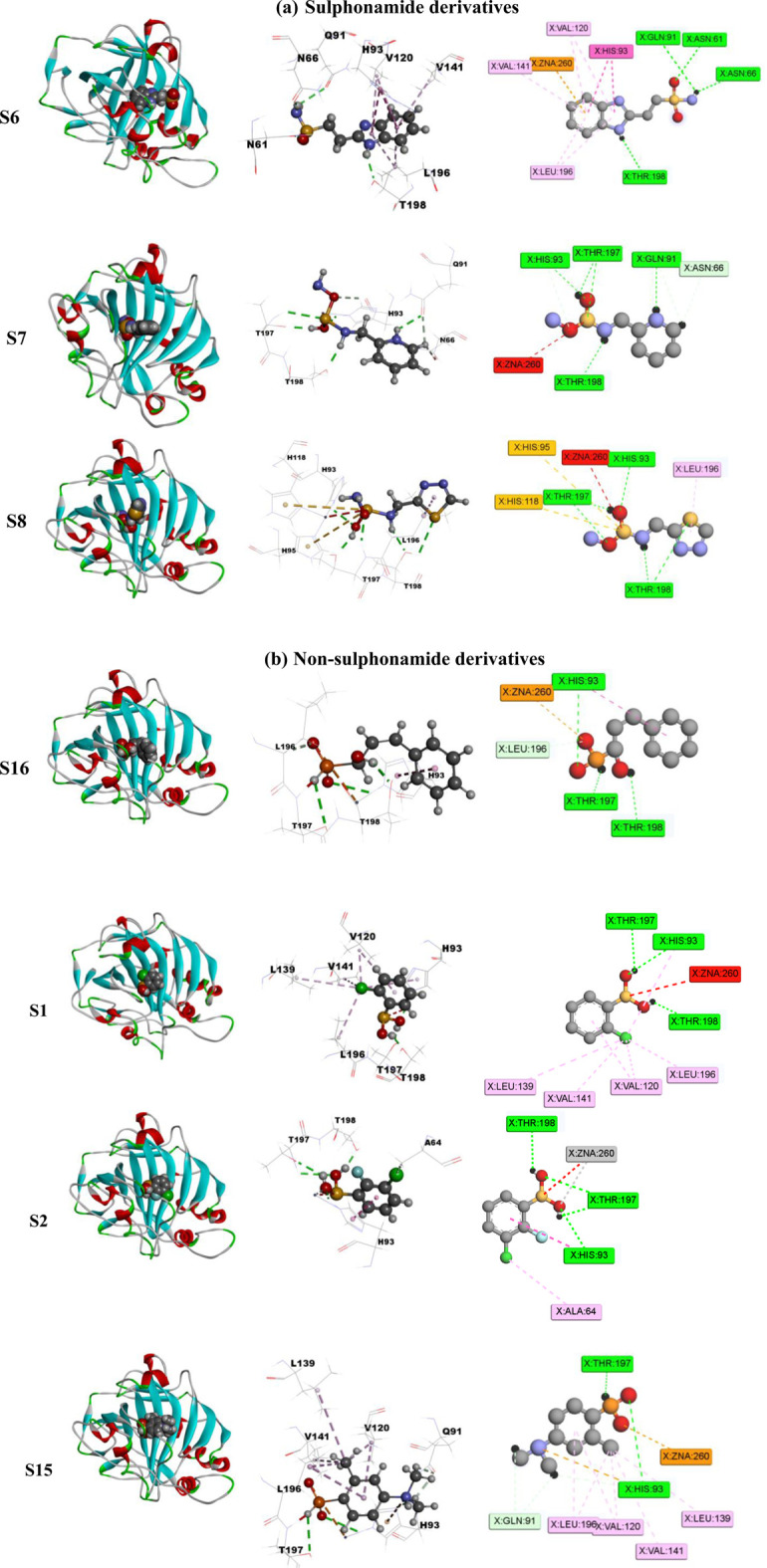
Schematic representing the docked interactions of various **(a)** sulphonamide and **(b)** non‐sulphonamide derivatives within the active site of CAII.

Screening of the generated library of small-molecule ligands (sulfonamide and non-sulfonamide) identified from the ZINC database was carried out to obtain deeper insight into the binding interactions with the active site of CAII via molecular docking investigations (Irwin et al., 2012). For this purpose, the GLIDE module of Schrödinger was employed. From the library, 186 ligands were shortlisted and ranked by their dock score (Supplementary Table S1; Supplementary Figure S3), and the top 10 ligands were considered for further investigations (Figure 9). Based on the values of dock score, the effective binding of the above ligands with CAII was ranked, and their binding modes were evaluated. The structural details of all the ligands are given in Supplementary Table S2. The pertinent results are given in Table 5. The binding site was mostly composed of polar residues such as *Lys131*, *Gln135*, and *Thr198*, non-polar amino acids like *Gly5*, *Pro199*, and *Val209*, and aromatic residues such as *Phe92*, *His121*, and *Phe94*. The list of binding site interacting residues is given in Table 6. To narrow the investigation, the top three ligands in each of the sulfonamide and non-sulfonamide derivatives were chosen for evaluating their dynamic interactions with CAII. A strong π–cation interaction was observed for all the selected ligands that were actively coordinated to the Zn^2+^ of CAII. In the case of non-sulfonamide ligands, interactions were observed with residues including *His93, Thr197*, and *Thr198* in addition to the observed π–alkyl interaction with *Val120*, *Leu139*, *Val141*, and *Leu196*. The sulfonamide ligands also exhibited strong hydrogen bonding interactions with *Asn61* and *Asn66* and π–sulfur interaction with *His93*. Moreover, the residues such as *Val120*, *Val141*, and *Leu196* formed a π–alkyl interaction with the phenyl ring of the ligands. The best docked poses of sulfonamide (S6, S7, and S8) and non-sulfonamide (S1, S2, S15, and S16) ligands are shown in Figure 9, while the remaining ligands are shown in Supplementary Figure S1.

**TABLE 5:** Evaluated values of dock score and free energy parameters (kcal mol^−1^) for the binding of various ligands with CA2.

Inhibitor	Dock score	∆G_Bind_	∆G_Bind_						
			Coulomb	Covalent	H-bond	Lipo	Packing	Solv	vdW
S1	−8.004	−46.28	−54.92	0.79	−1.90	−7.55	−2.28	38.8	−19.21
S2	−7.991	−40.97	−47.30	2.63	−1.63	−8.11	−2.22	35.13	−19.47
S3	−7.726	−45.51	−57.84	0.75	−1.88	−4.39	−2.36	38.53	−18.33
S4	−7.289	−42.65	−49.56	2.63	−1.65	−7.04	−2.16	33.19	−18.06
S5	−7.189	−43.12	−46.29	2.78	−1.65	−8.99	−2.04	29.71	−16.64
S6*	−6.920	−27.44	−18.74	2.73	−2.16	−8.55	−3.37	28.45	−25.8
S7*	−6.850	−40.31	−90.94	3.33	−2.25	−2.34	−1.53	72.92	−19.51
S8*	−6.720	−39.84	−53.99	1.27	−2.15	−6.45	−1.06	47.71	−25.16
S9*	−6.720	−24.88	−12.71	−1.08	−1.92	−10.39	−1.19	25.12	−22.71
S10*	−6.710	−43.44	−80.13	3.08	−2.81	−1.57	−2.33	61.29	−20.97
S11*	−6.72	−26.02	−25.07	4.45	−2.15	−6.02	−2.12	27.92	−23.04
S12	−6.102	−14.4	30.75	2.04	−1.41	−12.59	−1.02	−5.89	−26.28
S13	−5.298	−6.18	47.87	2.07	−1.76	−7.83	0	−19.93	−26.6
S14	−4.964	−0.51	44.17	0.53	−1.51	−3.28	0	−21.31	−19.12
S15	−7.909	−6.94	23.37	0.71	−0.34	−6.95	−2.01	2.57	−24.3
S16	−8.284	−11.18	17.66	3.3	−1.36	−11.2	−1.1	2.28	−20.76
S17*	−6.092	−21.02	−8.12	0.86	−0.58	−14.11	−1.17	26.26	−24.17
S18	−5.968	−15	53.83	1.78	−1.88	−11.45	−1.03	−30.21	−26.04
S19	−5.718	−23.11	−14.92	2.13	−1.3	−11.48	−1.38	26.93	−23.1
S20	−5.683	−36.27	−9.69	0.36	−1.06	−12.14	−0.95	12.5	−25.29

*Sulfonamide derivatives.

**TABLE 6:** Interacting residues of CAII with various ligands.

Ligand type	Ligand#	Interacting residue
Sulfonamide	S6	*N61*, *N66*, *Q91*, *H93*, *V120*, *V141*, *V196*, and *T198*
	S7	*N66*, *Q91*, *H93*, *T197*, and *T198*
	S8	*H93*, *H95*, *H118*, *L196*, *T197*, and *T198*
Non-sulfonamide	S1	*H93*, *V120*, *L139*, *V141*, *L196*, *T197*, and *T198*
	S2	*A64*, *H93*, *T197*, and *T198*
	S15	*Q91*, *H93*, *V120*, *L139*, *V141*, *L196*, and *T197*
	S16	*H93*, *L196*, *T197*, and *T198*

The scores from various free energy parameters that contribute significantly to the overall binding free energy of the docked interactions are given in Table 5. For example, the van der Waals contribution captures the close contact and steric interactions between the ligand and receptor. A good packing contribution is recognized with a significantly negative score. Favorable hydrophobic interactions depict the exposure of the ligands inside buried pockets. Good packing of hydrophobic groups in hydrophobic environments is energetically favored. These interactions reduce solvent exposure for non-polar atoms. This also mimics the free energy gain from hydrophobic packing.

The various residues of CAII that were effectively involved in the binding interactions with the ligands are given in Table 6. The binding pocket is mainly composed of polar residues like *Asp*, *Thr*, and *Gln* and non-polar residues, including *Val*, *Leu*, and Ala. Based on the dock score, sulfonamide ligands S6, S7, and S8 and non-sulfonamide ligands S1, S2, S15, and S16 were ranked sequentially. Among all the shortlisted ligands, S6 was observed to be exceptional with regard to its interaction with Zn^2+^. In this case, a strong π–cation interaction was observed with the aromatic ring, in addition to multiple strong hydrogen bonding interactions with *Asn61*, *Asn66*, *Gln91*, and *Thr198*. The benzimidazole moiety of the ligand was involved in π–π interaction with His93, while residues *Val120*, *Val141*, and *Leu196* were involved in π–alkyl interactions. With all other ligands (S7 and S8), coordinate bonding interactions were observed with the sulfonamide group of the ligands and the Zn^2+^ of CAII (Figure 9). In S7, the interactions were contributed through four strong conventional hydrogen bonds with *Gln91*, *His93*, *Thr197*, and *Thr198*. In S8, *Asn66*, *Gln91*, and *Thr198* formed three strong hydrogen bonds with the sulfonamide moiety, while interactions were observed with the Zn^2+^ and the sulfonamide group in addition to the π cloud of the ligand. Other interactions, like π–sulfur and π–alkyl interactions, were also involved in the stabilization.

In the case of non-sulfonamide ligands, Zn^2+^ was strongly coordinated to the –SO_2_-NH- moiety (Figure 9). In all the cases, strong hydrogen bonds were observed with residues *His93*, *Thr197*, and *Thr198*, in addition to other interactions like π–π and π–alkyl interactions. The free energy parameters associated with the best docking interactions are given in Table 5 (Supplementary Table S1).

### Molecular dynamics simulations

Although the docking investigations have revealed the effectiveness of binding interactions, the influence of dynamic motions under physiological conditions was not taken into account during the evaluation. Therefore, further investigations into the stability of interactions were carried out with the help of molecular dynamics simulations. These interactions are not static, and it is necessary to evaluate the variations in free energy and stability through dynamic simulations. For the purpose of understanding the dynamic behavior of the bio-molecular systems at their atomistic levels, molecular dynamics simulations were performed for the selected protein–ligand complexes. A detailed analysis of the key dynamic parameters, including the root mean square deviation (RMSD), radius of gyration (Rg), root mean square fluctuation (RMSF), hydrogen bonding, and solvent-accessible surface area (SASA), was carried out for the CAII protein and its interactions with sulfonamide and non-sulfonamide ligands. Here, the stability of the complexes under dynamic environments was evaluated for a period of 100 ns.

### Influence of ligand binding to the active site of CAII as shown by RMSD

**FIGURE 10:**
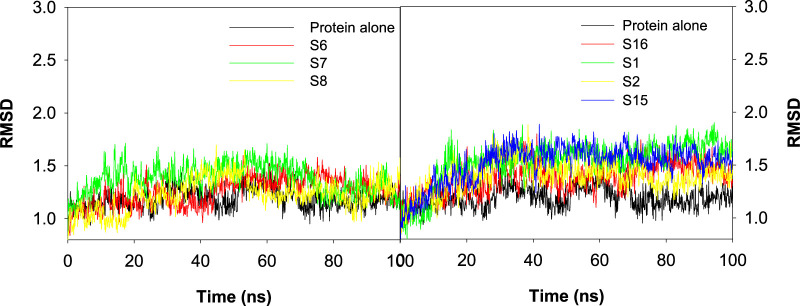
Plot depicting the RMSD values for the best docked poses of sulfonamide (S6, S7, and S8) and non-sulfonamide (S1, S2, S15, and S16) ligands of CAII.

**FIGURE 11:**
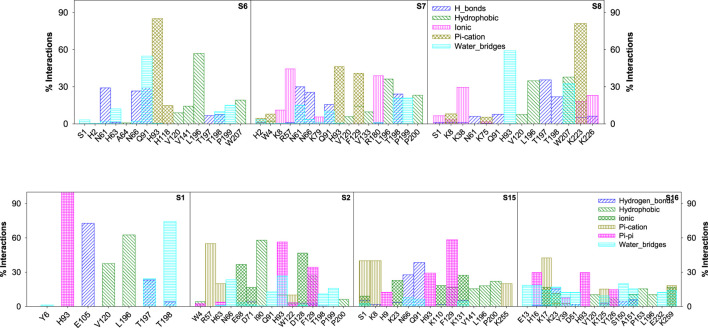
Bar graph depicting the percentage contribution from each amino acid residue of CAII toward various stabilizing interactions with sulfonamide and non-sulfonamide derivatives.

The root mean square deviation (RMSD) was evaluated as a measure of the influence of various ligands on the structural integrity of CAII and to quantify the changes incurred in the protein backbone throughout the simulation period. No significant changes were observed in the RMSD of the protein upon the approach of various best docked sulfonamide and non-sulfonamide ligands. The results revealed that the system under investigation is more stable during the interaction period. RMSD evaluations for the protein in the presence and absence of ligands were compared while assessing the stability of the complexes. The RMSD trajectories for various protein–ligand complexes are depicted in Figure 10. The average RMSD values for the complexes of S6, S7, and S8 (sulfonamides) were 1.26 ± 0.38 Å, 1.38 ± 0.37 Å, and 1.23 ± 0.46 Å, and the average RMSD values for S1, S2, S15, and S16 (non-sulfonamides) were 1.53 ± 0.56 Å, 1.39 ± 0.55 Å, 1.51 ± 0.69 Å, and 1.38 ± 0.69 Å respectively. The fluctuations observed in the trajectory during the initial simulation period were ignored as they may contribute to the stabilization phase, where the protein–ligand complex adjusts from its starting structure toward a thermodynamically favorable conformation under the field of applied force. These early phases include the relaxation of steric clashes, redistribution of solvent molecules, and adjustment of functional group orientations for meaningful structural deviations of the equilibrated complex (Grossfield et al., 2018; Schreiner et al., 2012; Walton and Vanvliet, 2006). Detailed analyses of the trajectories have shown that several factors and forces influence the stability of interactions, including solvent bridges, hydrophobic, π–alkyl, and hydrogen bonding*.* The extents of various interactions that contribute to the stability are depicted in Figure 11.

The various types of stabilizing interactions that influenced the binding of different ligands to the binding cavity, along with their percentage contributions, are depicted in Figure 11. In the case of S6, major stabilization was observed from the hydrophobic and π–π interactions involving residues like *Val120*, *Val141*, *Leu196*, *Trp207* (hydrophobic), and *His93* and *His118* (π–π), although hydrogen bonding and water bridges also showed significant stabilizations. For S7, *Asn61*, *Arg57*, *His93*, and *Thr198* revealed maximum stabilizing contributions through hydrogen bonding, hydrophobic forces, ionic forces, π–π forces, and solvent bridges, respectively. In S8, residues including *Thr197*, *Leu196*, *Lys38*, *Lys223*, and *His93* were involved in hydrogen bonding, hydrophobic forces, ionic forces, and π–cation forces, and solvent bridge interactions, respectively.

In the case of non-sulfonamide ligands, except S1, other ligands revealed significant contributions through π–cation interactions. Residues like *His93*, *Glu105*, *Leu196*, and *Thr198* showed significant contributions through π–π forces, hydrogen bonding, hydrophobic forces, and solvent bridges, respectively. The % contributions with other ligands are depicted in Figure 11.

### Impact of the ligand–CAII interaction on RMSF

**FIGURE 12:**
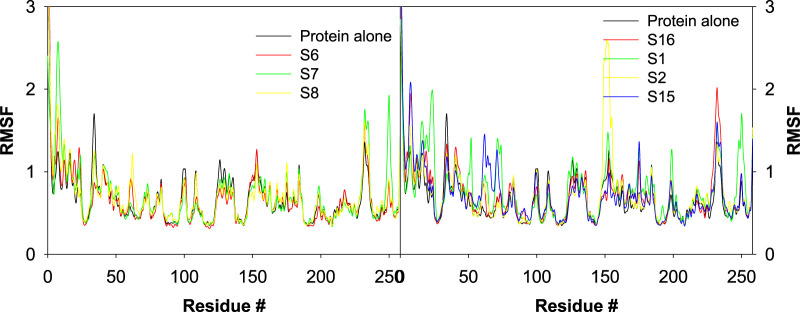
Plot depicting the trajectory of RMSF for the best docked poses of sulfonamide (S6, S7, and S8) and non-sulfonamide (S1, S2, and S3) ligands of CAII.

RMS fluctuations are another parameter to evaluate the dynamic stability of the complexes. In this study, the positional fluctuations of all amino acid residues of the selected protein–ligand complexes were observed and are depicted in Figure 12. There was no notable fluctuation observed for the selected complexes except for S2 and S7. *His2*, *His3*, *Lys8*, *His9*, *Asn10*, *Gly233*, *Gln234*, *Pro235*, and *Lys258* showed fluctuations >2 Å, and most of the residues that interacted with the ligand were located inside or close to the binding pocket. While analyzing the trajectory of the protein in the presence and absence of any ligand, only minor fluctuations were observed across all residues, with an average RMSF of 0.681 Å ± 2.22 Å. The average RMS fluctuations was observed for complexes S1, S2, S3, S6, S7, S8, S11, S12, S13, S14, S15, and S16 were 0.78 ± 1.24 Å, 0.743 ± 1.92 Å, 0.86 ± 2.34 Å, 0.67 ± 2.72 Å, 0.71 ± 1.08 Å, 0.71 ± 2.49 Å, 0.70 ± 1.14 Å, 0.68 ± 2.72 Å, 0.70 ± 2.13 Å, 0.67 ± 2.48 Å, 0.73 ± 2.61 Å, and 0.73 ± 2.25 Å, respectively.

### Influence of various ligand interactions with CAII on radius of gyration (Rg)

**FIGURE 13:**
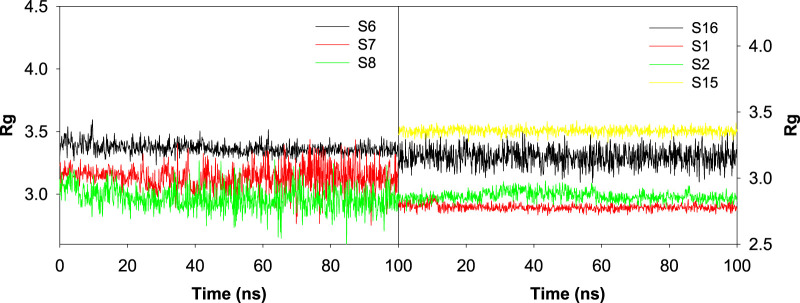
Plot depicting the trajectory of the radius of gyration for the best docked sulfonamide (S6, S7, and S8) and non-sulfonamide (S1, S2, S15, and S16) ligands upon their interaction with CAII.

The influence of various ligands on the stability of the complexes was evaluated by analyzing the radius of gyration during the 100 ns contact time. This helps to assess the protein’s structural compactness during interaction with different ligands. Rg is used to calculate the distance between the ligand’s central axis and the atom’s rotational position at which the most energy was transferred. It is well known that the Rg values are influenced by the conformational changes that are brought about by the binding of various ligands to the target. Here, the values of Rg for all the selected protein–ligand complexes were examined. Figure 13 revealed the trajectory of Rg during the evaluation period. The average radius of gyration was found to be 2.78 ± 0.11 Å, 2.86 ± 0.16 Å, 2.83 ± 0.16 Å, 3.37 ± 0.24 Å, 3.31 ± 0.61 Å, 2.97 ± 0.57 Å, 3.12 ± 0.37 Å, 2.82 ± 0.16 Å, 3.01 ± 0.26 Å, 2.45 ± 0.10 Å, 3.36 ± 0.13 Å, and 3.16 ± 0.30 Å, respectively, for the S1, S2, S3, S6, S7, S8, S11, S12, S13, S14, S15, and S16 complexes.

### Influence of various ligand interactions with CAII on solvent-accessible surface area (SASA)

**FIGURE 14:**
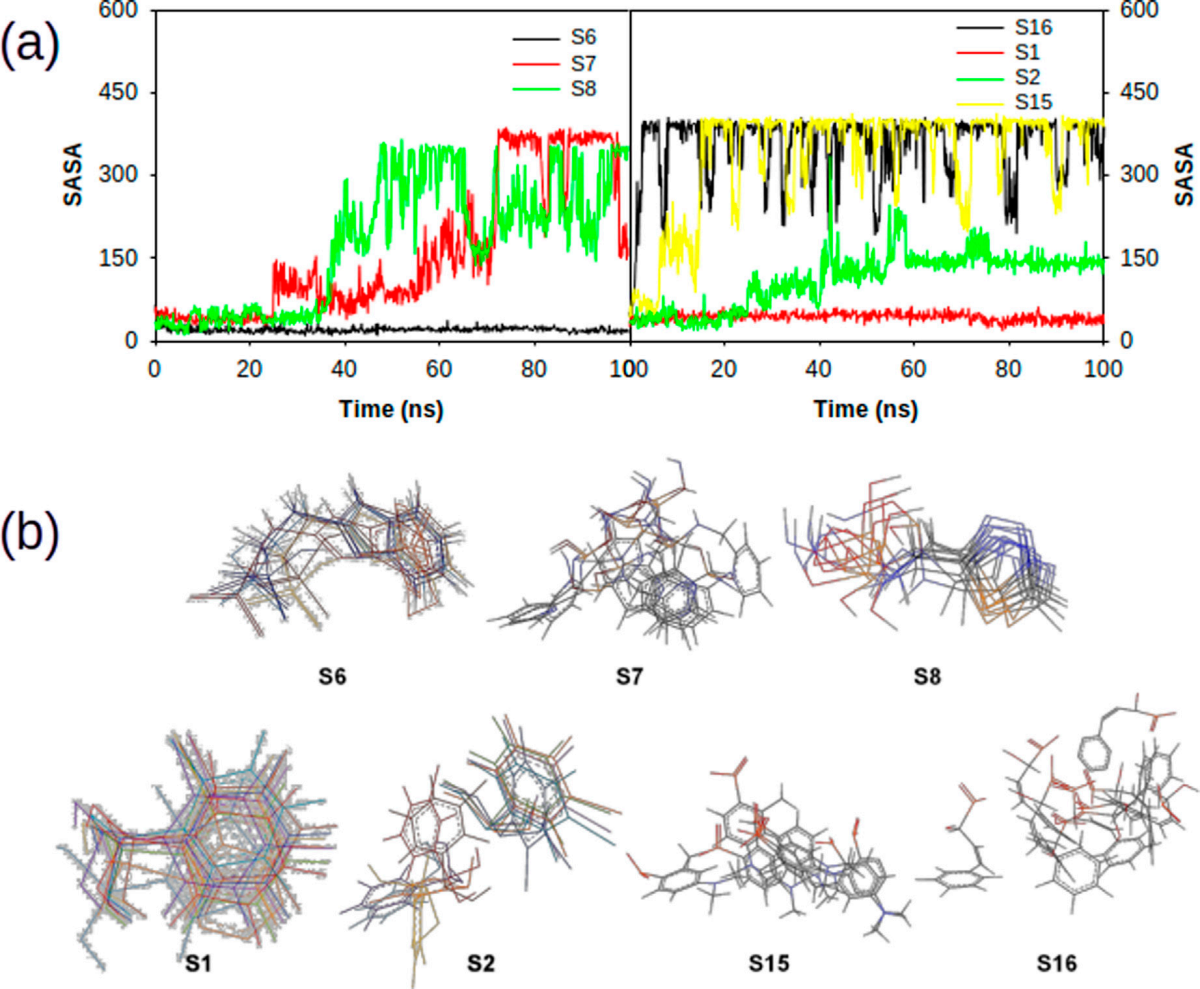
**(a)** Plot depicting the trajectory of solvent accessible surface area of various sulphonamide (S6, S7 and S8) and non-sulphonamide (S1, S2, S15 and S16) ligands upon its interaction with CAII. **(b)** 2-dimensional overlaid alignment of corresponding ligands at various time points during the course of molecular dynamic simulations.

The interaction of ligands with CAII has a significant impact on the characteristics of the polar and non-polar surfaces of the ligand. The SASA analysis helps to locate the crucial binding pocket residues on the protein target and evaluate their accessibility to solvent molecules. Figure 14 shows the SASA trajectory. Supporting the earlier observations, the SASA trajectories also showed that non-sulfonamide ligands interact more strongly than sulfonamide derivatives, although significant fluctuations were detected at multiple points over a 100 ns simulation period. The position of the ligands at various time points during the course of simulation is also depicted in Figure 14. The observed fluctuations could be due to the presence of freely rotatable phenyl moieties on non-sulfonamide ligands rather than sulfonamide ligands. The average SASA for S1, S2, S3, S6, S7, S8, S11, S12, S13, S14, S15, and S16 complexes was found to be 42.91 ± 33.14 Å, 111.87 ± 269.44 Å, 37.53 ± 55.66 Å, 20.21 ± 51.16 Å, 159.48 ± 294.18 Å, 242.83 ± 177.80 Å, 125.66 ± 310.71 Å, 51.95 ± 71.81 Å, 213.89 ± 307.96 Å, 71.39 ± 274.949 Å, 333.46 ± 218.05 Å, and 354.87 ± 201.508 Å, respectively. The data from SASA assessments suggested that the solvent significantly contributes to stabilizing ligands as they bind to the cavity.

### Influence of various ligand interactions with CAII on hydrogen bonding

**FIGURE 15:**
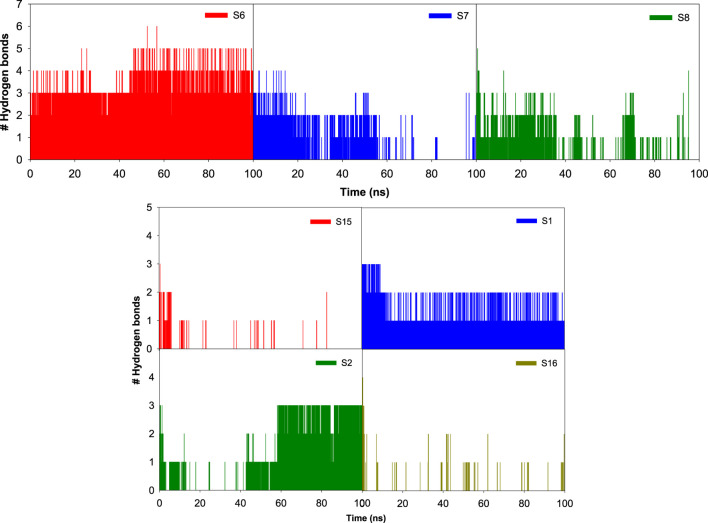
Bar graph depicting the number of hydrogen bonds between various sulfonamide (S6, S7, and S8) and non-sulfonamide (S1, S2, S15, and S16) ligands with the different binding pocket residues of CAII, along with their respective hydrogen bonding potential surface.

**FIGURE 16:**
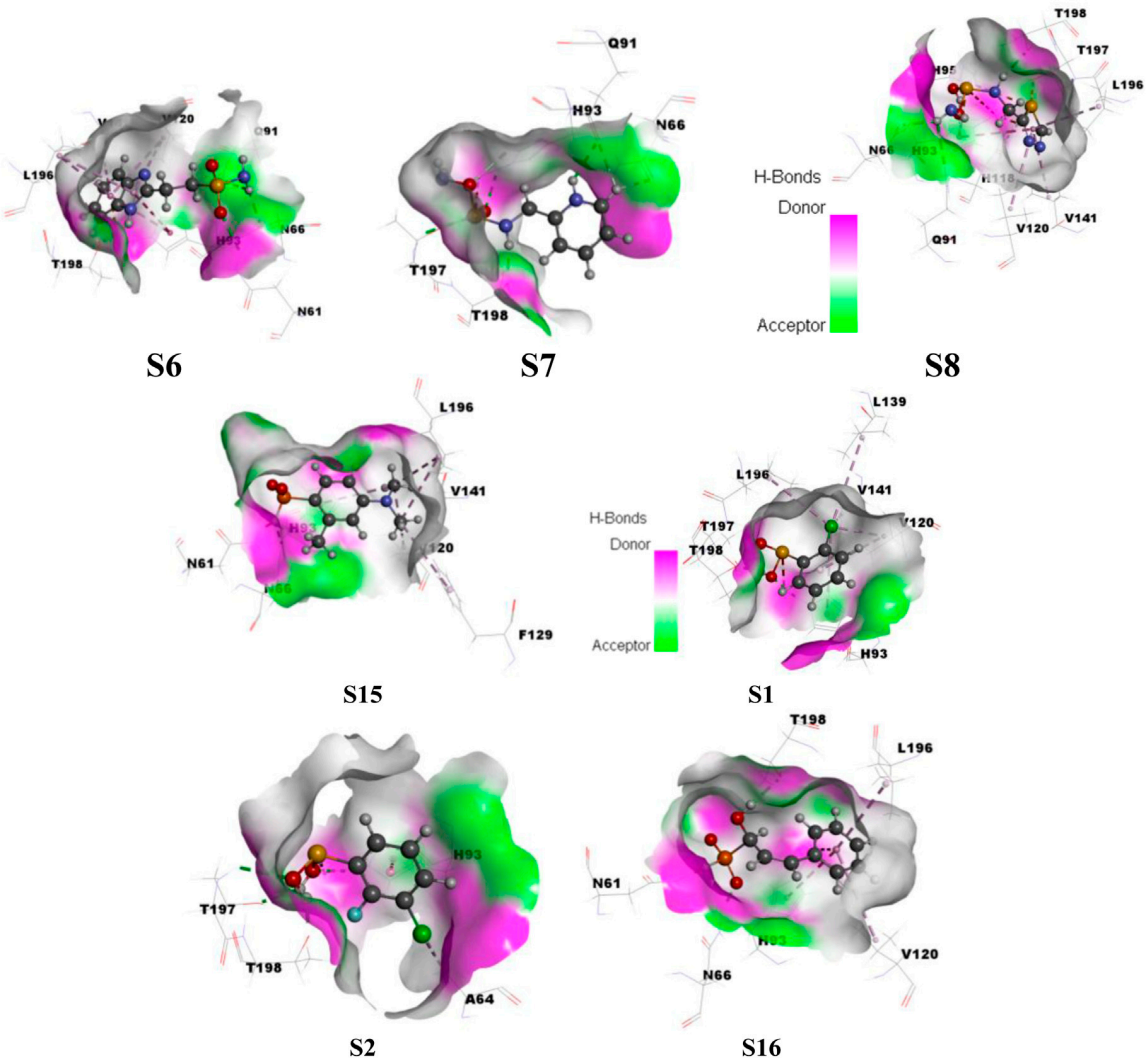
Surface depicting the hydrogen bonding potential for the various sulfonamide (S6, S7, and S8) and non-sulfonamide (S1, S2, S15, and S16) ligands toward their interaction with CAII.

A variety of interactions preserve the structural integrity and stability of biomolecules. Among all the interactions, hydrogen bonding plays a major role in stabilizing the protein–ligand complexes. Here, investigations on the hydrogen bonding interactions were carried out for 100 ns, and the results are depicted in Figure 15. The factors responsible for stability are listed in the RMSD results. Among them, the hydrogen bonding contribution is greater because comparatively more energies are involved in binding. The results showed that the lifetime of the hydrogen bond indicates its strength. Several weak interactions, like hydrogen bonding and van der Waals interactions, are involved in energy transfer and enhance stability. The ability of various ligands to interact with the binding pocket residues of CAII is represented by their potential surface, as shown in Figure 16.

### Evaluation of pharmacokinetic properties: ADME

The above evaluated ligands were further investigated for their druggability through their pertinent pharmacokinetic characteristics, including absorption, distribution, metabolism, and excretion. The QikProp module of Schrödinger was utilized for the purpose. As a preliminary evaluation, Lipinski’s rule of five was taken into account along with star, central nervous system, human oral absorption, solvent accessibility, *etc.*

The star value represents the degree to which the ADME characteristics of a drug differ from those of 95% of other known inhibitors. The number of non-trivial, non-hindered rotatable bonds is represented as rotors (6.0–15.0). The CNS score ranges from −2 to +2, which influences ADME processes by regulating drug absorption through the blood–brain barrier, distribution via selective transport mechanisms, metabolism by brain-specific enzymes, and excretion through cerebrospinal fluid and efflux transporters. Molecules with a human oral absorption (HOA) value exceeding two are considered strong candidates for drug development. Lipinski’s rule of five plays a crucial role in ADME by providing guidelines to predict a compound’s oral bioavailability based on its molecular properties, including molecular weight (130.0–725.0), number of hydrogen bond donors (0.0–6.0), number of hydrogen bond acceptors (2.0–20.0), octanol water coefficient (log P_o/w_ = −2.0–6.5), and solubility (log S = −6.5 to 0.5), which collectively influence absorption and distribution characteristics. In ADME, solvent-accessible surface area (SASA) falls within the range of 300–1000, which is crucial as it influences a drug’s solubility, permeability, and interaction with biological membranes and proteins, thereby affecting its absorption and distribution properties. Polrz refers to the distribution of electric charge within a molecule and can influence a drug’s solubility, permeability through biological membranes, and interactions with proteins, which are critical factors in its ADME properties (polrz = 13.0–70.0). Hexadecane/gas coefficient is represented by log P_C16_ (4.0–18.0). logHERG predicted IC_50_ value represents the concentration at which a substance inhibits HERG K^+^ channels (log HERG = below −5). PCaco helps predict a compound’s permeability through the intestinal epithelium, indicating its potential oral bioavailability and absorption by assessing the five Lipinski’s rule properties (<25 poor, >500 great). Log BB value denotes the predicted brain/blood partition coefficient (−3.0 to 1.2). From the above evaluations, the compounds S1, S2, S3, S7, S11, and S14 did not show favorable pharmacokinetic properties.

The skin permeability coefficient is determined in terms of logK_p_ (−8 to −1). The water–gas partition coefficient should lie between 4 and 45. The drug binding with human serum albumin was determined in terms of logK_HSA_, and it ranges between −1.5 and 1.5 (Colmenarejo, 2003). Apart from the above-mentioned exceptions, all the other compounds have revealed their drug potential. The above parameter values are tabulated in Table 7. The ZINC database compounds that satisfy all criteria were utilized for subsequent stages of docking and MM-GBSA studies. The relevant data pertaining to the toxicity of the reported derivatives, along with a positive control acetazolamide, are compiled in Table 8 (Supplementary Table S5) (ref: International Immunopharmacology, 134, 112,178. https://doi.org/10.1016/j.intimp.2024.112178).

**TABLE 7:** Pharmacokinetic properties as evaluated from absorption, distribution, metabolism, and excretion (ADME) investigations.

Molecule	Star	Rotor	Central nervous system	Molecular weight	Donor hydrogen bond	Acceptor hydrogenbond	Octanol ‐ water partition coefficient	Aqueous solubility	Inhibition of HERG channels	permeability coefficient	Blood–brain–barrier	Skin permeability coefficient	Predicted human oral absorption
S1	0	2	−1	176.617	1	4	9.793	1.786	−1.736	14.935	−0.004	−2.53	58.416
S2	0	2	0	194.608	1	4	1.678	−1.13	−1.746	13.542	−0.011	−2.53	57.023
S3	0	2	−1	194.608	1	4	1.695	−1.2	−1.784	12.154	−0.005	−2.67	56.286
S6	0	4	−1	199.64	3	3	−0.73	−1.45	−2.73	28.43	−0.27	−5.32	48.72
S7	0	4	−2	225.27	3	6	0.14	−1.99	−4.58	188.48	−1.28	−3.8	68.51
S8	0	4	−1	193.23	2	6	0.95	−1.57	−3.83	408.9	−0.84	−7.88	78.58
S11	0	3	−2	212.22	2	6	−0.16	−1.9	−3.69	203.75	−1	−3.9	67.35
S12	0	4	−1	179.218	3	3	−0.84	−1.1	−2.711	35.317	−0.33	−5	49.723
S13	0	3	1	189.272	2	4	−0.74	−1.63	−2.083	62.28	0.003	−5.2	54.741
S14	1	1	1	143.185	2	3.5	−1.45	−0.73	−1.28	62.926	0.123	−5.4	50.63
S15	0	3	−1	199.189	2	6	1.232	−1.9	−1.91	334.307	−0.3	−2.6	79.335
S16	0	5	−1	198.158	3	6.7	0.615	−1.5	−2.528	127.075	−0.8	−2.8	68.204

**TABLE 8:** Toxicity parameters as evaluated for various sulfonamide and non-sulfonamide ligands.

Target	Acetazolamide	S1	S2	S3	S6	S7	S8	S11	S12	S13	S14	S15	S16
Predicted LD_50_ (mg/kg)	4300	1190	500	500	49	1700	1000	1829	2400	1600	5490	1345	2500
Predicted toxicity class	5	4	4	4	2	2	4	4	5	4	6	4	5
Hepatotoxicity	0.56	0.69	0.80	0.80	0.63	0.61	0.58	0.54	0.62	0.65	0.81	0.65	0.72
Carcinogenicity	0.51	0.62	0.84	0.84	0.50	0.54	0.61	0.54	0.68	0.53	0.58	0.55	0.85
Immunotoxicity	0.99	0.96	0.98	0.95	0.96	0.93	0.97	0.52	0.62	0.57	0.51	0.63	0.58
Mutagenicity	0.85	0.97	0.88	0.88	0.59	0.50	0.50	0.88	0.56	0.55	0.59	0.55	0.60
Cytotoxicity	0.54	0.93	0.80	0.80	0.68	0.60	0.60	0.86	0.53	0.54	0.62	0.68	0.64

Based on the values of star, molecular weight, hydrogen bonding capacity, and number of rotational bonds, the drug likeness is predicted as all the molecules to possess good drug-likeness (Star = 0), except S14. The hydrogen bond donor/acceptor values are within standard ranges, with S16 having the highest count with 3 donors and 6.7 acceptors. From the values of log Po/w, S1 is observed to be extremely lipophilic (9.793), which will have a significant influence on the solubility and permeability, while S14 is more hydrophobic (−1.45). All the molecules except S1 (1.786) have moderate aqueous solubility. All the molecules have a low risk of cardiotoxicity, although S7 and S8 are high. The intestinal permeability is also in range for all the molecules, while S8 and S15 reveal excellent permeability. S8 and S15 have good oral absorbability, while S6 and S12 show a lower probability of absorption. S2, S3, S13, and S14 display potent CNS activity. S14 and S13 have potent blood–brain barrier (BBB) penetration, while others do not effectively cross the BBB. Most of the compounds show low dermal permeability, with S8 being the least permeable and S1 and S2 being the most permeable. Based on the above observations, it can be concluded that S8 (sulfonamide) and S15 (non-sulfonamide) are the best overall candidates with high oral absorption, good permeability, and moderate toxicity risk. S14 and S13 have favorable BBB penetration and CNS scores. Compounds S6, S11, and S12 have moderate PHOA and low permeability. Because all the compounds present unique profiles, they may suit different therapeutic needs. Further *in vitro* and *in vivo* validations are recommended to confirm these predictions.

ProTox-II, an online browser-based toxicology prediction program, was utilized to predict the toxicity targets for the different ligands (Banerjee et al., 2018). Acute toxicity, toxicity endpoints, organ toxicity, stress response pathways, nuclear receptor signaling pathways, and other toxicity endpoints were all predicted. For validation, this tool uses selective oversampling in conjunction with the probability-based CLUSTER cross-validation. This tool was used to assess the possible risks, namely, the immunotoxicity, mutagenicity, cytotoxicity, carcinogenicity, and hepatotoxicity, associated with different sulfonamide and non-sulfonamide ligands. The predicted toxicity parameters for the shortlisted ligands are given in Table 8.

The results indicate that most compounds exhibit moderate toxicity profiles, with predictions varying from high acute toxicity for S6 to low acute toxicity for S14. Correspondingly, S6 and S7 fall into class-2 toxicity (more toxic), while S14 falls into class 6 (least toxicity). Compounds S12, S14, and S16 show relatively low hepatotoxicity and carcinogenicity probabilities, whereas S2 and S3 have higher values (≥0.80), suggesting a greater risk for liver damage and cancer potential. High immunotoxicity and mutagenicity scores are observed for most compounds (especially S1–S3), with S6 being comparatively lower in mutagenicity.

Receptor-based toxicity predictions indicate that nearly all compounds interact strongly with nuclear receptors, particularly AR, ER, PPARγ, and AhR, suggesting potential endocrine or metabolic pathway interactions. Notably, compounds also display significant activation of stress response elements, such as Nrf2/ARE and HSE, which may imply oxidative or heat shock stress responses. Mitochondrial toxicity, indicated by mitochondrial membrane potential (MMP) values, is highest for S11 and S12. Finally, most compounds show strong interactions with tumor suppressor p53 and DNA damage-associated protein ATAD5, hinting at possible genotoxic stress.

Structurally, the higher toxicity of S6 and S7 could be attributed to their heteroaromatic-rich sulfonamide scaffolds. S6 bears a benzimidazole–sulfonamide framework that furnishes an extended, planar π-surface and basic nitrogens, promoting off-target π–cation–π contacts and cationic amphiphilicity, consistent with its class-2, very low LD_50_ value. Meanwhile, S7 exhibits the highest H-bond acceptor load (6) and multiple donors (3), which can increase interaction promiscuity and align with the increased risk for hERG as observed *in silico*; together these features rationalize its class-2 acute toxicity assignments. In contrast, S8 maintains the sulfonamide Zn-binding pharmacophore without these liabilities and yields class-4 toxicity and a more favorable ADME/toxicity profile. Therefore, S6 and S7 present the highest toxicity concerns due to their low LD_50_ and toxicity class, while S16 appear the safest based on acute toxicity, hepatotoxicity, and lower receptor activation profiles.

## Conclusion

Following a fragment-based drug discovery approach, the potential of a series of small-molecule inhibitors—sulfonamide and non-sulfonamide derivatives for human carbonic anhydrase II—was evaluated via computational molecular docking and molecular dynamics simulations. Initial screening of the library was carried out by evaluating their pharmacophoric properties to evaluate their relative druggability. Sulfonamide derivative S8 and non-sulfonamide derivatives S15 and S16 were found to be potent CAII inhibitors. It is also demonstrated that non-sulfonamide scaffolds can achieve strong and selective CAII inhibition, expanding beyond the classical sulfonamide framework. Further evaluations on the molecular interactions have revealed that the nature and size of the functional groups of the proposed inhibitors that interact with the catalytic Zn^2+^ and surrounding amino acid residues play crucial roles in disseminating energy parameters. It also signifies the role of stable Zn^2+^ coordination and persistent interactions with *His93*, *Leu196*, *Thr197*, and *Thr198*, which form a molecular basis for their inhibitor potency. In all cases, the central Zn^2+^ ion has shown interaction with the ligand inhibitors via their π electron cloud, metal–carbon bond, electrostatic interactions, *etc*., in addition to conventional hydrogen bonding, van der Waals, and π–π interactions. The potential energy surface scans have revealed that angular motions of the functional groups display weak destabilizing effects on the molecular twisting (dihedral angle) of the functional derivatives. The pharmacodynamics evaluation has shown the possible drug characteristics of these molecules. Among all the ligands, S1–5 and S16–17 have shown promising inhibitory properties based on their dock score values. Further dynamic simulations have revealed that non-sulfonamides have slightly higher average RMSD (1.45 Å) than sulfonamides (1.29 Å). In the case of sulfonamides, π–cation interactions were dominant, while in non-sulfonamides, π–π interactions were dominant. Similarly, large fluctuations were observed with *His2*, *His3*, *Lys8*, *His9*, *Asn10*, *Gly233*, *Gln234*, *Pro235*, and *Lys258* residues of CA, while no major destabilizing effects were observed through ligand interactions. From the above results, compounds S6, S7, and S8 (among sulfonamides) and S1, S2, S15, and S16 (among non-sulfonamides) have revealed their inhibition potential. Furthermore, the pharmacokinetic evaluations have shown deviations from the acceptable range in their ADME properties for S1–3, S7, S11, and S14, while toxicity evaluations have shown unacceptable toxicities for S2, S3, S6, S7, S11, and S12, which cannot be used as drugs. With all the above results, it can be concluded that S8 (sulfonamide) and S15 and S16 (non-sulfonamide) are potent molecules with appropriate inhibition potential for human CAII. Moreover, this study provides atomistic-level guidance for rational modifications of CAII inhibitors, further enabling the design of next-generation molecules with improved potency and selectivity.

## Data Availability

The original contributions presented in the study are included in the article/Supplementary Material, further inquiries can be directed to the corresponding author.
