# Supplementary material for: Computational insights into novel inhibitors: virtual screening of small molecules against human carbonic anhydrase II
Source: Front Chem. 2025 Oct 2;13:1627793. doi: 10.3389/fchem.2025.1627793 (PMC12528100; doi:10.3389/fchem.2025.1627793)
Supplement: Supplementary file 1 [file DataSheet1.docx]

**Computational Insights into Novel Inhibitors: Virtual Screening of Small Molecules against Human Carbonic Anhydrase II**

**Sermarajan Arunachalam, Balamurali MM* and R[amachandran Gnanasekaran*](https://www.sciencedirect.com/science/article/pii/S109332631730671X#!)**

**Department of Chemistry, School of Advanced Sciences**

**Vellore Institute of Technology, Chennai Campus, Chennai 600127**

**Table S1.** Dock score and free energy parameters for various proposed inhibitors of human CAII as evaluated by molecular docking.

|  | **Dock Score** | **∆G_Bind_ (kcal mol^-1^)** | **∆G_Bind_ (kcal mol^-1^)** | | | | | | |
| --- | --- | --- | --- | --- | --- | --- | --- | --- | --- |
|  |  |  | **Coulomb** | **Covalent** | **H-Bond** | **Lipo** | **Packing** | **Solv** | **VdW** |
| S1 | -8.004 | -46.28 | -54.92 | 0.79 | -1.9 | -7.55 | -2.28 | 38.8 | -19.21 |
| S2 | -7.991 | -40.97 | -47.3 | 2.63 | -1.63 | -8.11 | -2.22 | 35.13 | -19.47 |
| S3 | -7.726 | -45.51 | -57.84 | 0.75 | -1.88 | -4.39 | -2.36 | 38.53 | -18.33 |
| S4 | -7.289 | -42.65 | -49.56 | 2.63 | -1.65 | -7.04 | -2.16 | 33.19 | -18.06 |
| S5 | -7.189 | -43.12 | -46.29 | 2.78 | -1.65 | -8.99 | -2.04 | 29.71 | -16.64 |
| S6 | -6.92 | -27.44 | -18.74 | 2.73 | -2.16 | -8.55 | -3.37 | 28.45 | -25.8 |
| S7 | -6.85 | -40.31 | -90.94 | 3.33 | -2.25 | -2.34 | -1.53 | 72.92 | -19.51 |
| S8 | -6.72 | -39.84 | -53.99 | 1.27 | -2.15 | -6.45 | -1.06 | 47.71 | -25.16 |
| S9 | -6.72 | -24.88 | -12.71 | -1.08 | -1.92 | -10.39 | -1.19 | 25.12 | -22.71 |
| S10 | -6.71 | -43.44 | -80.13 | 3.08 | -2.81 | -1.57 | -2.33 | 61.29 | -20.97 |
| S11 | -6.72 | -26.02 | -25.07 | 4.45 | -2.15 | -6.02 | -2.12 | 27.92 | -23.04 |
| S12 | -6.102 | -14.4 | 30.75 | 2.04 | -1.41 | -12.59 | -1.02 | -5.89 | -26.28 |
| S13 | -5.298 | -6.18 | 47.87 | 2.07 | -1.76 | -7.83 | 0 | -19.93 | -26.6 |
| S14 | -4.964 | -0.51 | 44.17 | 0.53 | -1.51 | -3.28 | 0 | -21.31 | -19.12 |
| S15 | -7.909 | -6.94 | 23.37 | 0.71 | -0.34 | -6.95 | -2.01 | 2.57 | -24.3 |
| S16 | -8.284 | -11.18 | 17.66 | 3.30 | -1.36 | -11.2 | -1.1 | 2.28 | -20.76 |
| S17 | -6.092 | -21.02 | -8.12 | 0.86 | -0.58 | -14.11 | -1.17 | 26.26 | -24.17 |
| S18 | -5.968 | -15 | 53.83 | 1.78 | -1.88 | -11.45 | -1.03 | -30.21 | -26.04 |
| S19 | -5.718 | -23.11 | -14.92 | 2.13 | -1.3 | -11.48 | -1.38 | 26.93 | -23.1 |
| S20 | -5.683 | -36.27 | -9.69 | 0.36 | -1.06 | -12.14 | -0.95 | 12.5 | -25.29 |
| S21 | -7.188 | -43.12 | -46.28 | 2.78 | -1.65 | -8.99 | -2.04 | 29.72 | -16.65 |
| S22 | -7.188 | 13.42 | 29.68 | 2.39 | -1.54 | -4.85 | 0 | 5.35 | -17.61 |
| S23 | -7.153 | 12.95 | 27.22 | 2.54 | -1.55 | -3.67 | 0 | 7.35 | -18.93 |
| S24 | -6.822 | 12.95 | 27.22 | 2.54 | -1.55 | -3.67 | 0 | 7.34 | -18.93 |
| S25 | -6.79 | 12.09 | 30.51 | 0.21 | -0.81 | -2.74 | 0 | 1.83 | -16.91 |
| S26 | -6.785 | 11.45 | 45.89 | 3.99 | -1.92 | -9.17 | -1.32 | -2.44 | -23.58 |
| S27 | -6.767 | 11.42 | 55.25 | -2.66 | -1.45 | -1.9 | 0 | -11.86 | -25.97 |
| S28 | -6.767 | 11.43 | 55.29 | -2.66 | -1.45 | -1.9 | 0 | -11.86 | -25.98 |
| S29 | -6.73 | 11.26 | 25.55 | 1.46 | -1.73 | -1.21 | 0 | 2.21 | -15.01 |
| S30 | -6.677 | 10.86 | 52.82 | 2.26 | -1.17 | -5.56 | 0 | -21.5 | -15.98 |
| S31 | -6.676 | 9 | 48.9 | 1.23 | -1.07 | -1.89 | 0 | -20.19 | -17.98 |
| S32 | -6.66 | 9 | 48.9 | 1.23 | -1.07 | -1.89 | 0 | -20.2 | -17.98 |
| S33 | -6.633 | 7.87 | 56.31 | 1.66 | -1.52 | -6.97 | -1.8 | -17.1 | -22.71 |
| S34 | -6.614 | 7.83 | 70.28 | 1.1 | -1.4 | -8.29 | -2.15 | -23.13 | -28.57 |
| S35 | -6.594 | 7.21 | 49.34 | 2.8 | -1.53 | -10.64 | -1.32 | -4.68 | -26.77 |
| S36 | -6.54 | 6.13 | 40.86 | 3.12 | -1.38 | -8.66 | -0.48 | -3.68 | -23.65 |
| S37 | -6.503 | 3.95 | 27.54 | 1.05 | -1.15 | -10.78 | -2.05 | 13.34 | -24.01 |
| S38 | -6.493 | -0.32 | 49.55 | 0.83 | -1.77 | -9.87 | -1.36 | -11.53 | -26.17 |
| S39 | -6.493 | 1.01 | 48.75 | 2.29 | -1.88 | -11.39 | -1.84 | -5.08 | -29.83 |
| S40 | -6.408 | -0.64 | 50.74 | 0.45 | -1.41 | -5.66 | 0 | -21.09 | -23.66 |
| S41 | -6.374 | -0.89 | 50.69 | 0.46 | -1.43 | -5.64 | 0 | -21.37 | -23.6 |
| S42 | -6.374 | -0.64 | 50.74 | 0.45 | -1.41 | -5.66 | 0 | -21.09 | -23.66 |
| S43 | -6.371 | -2.5 | 25.32 | 0.92 | -0.8 | -5.62 | -2.34 | 0.23 | -20.2 |
| S44 | -6.371 | -0.89 | 50.69 | 0.46 | -1.43 | -5.64 | 0 | -21.37 | -23.6 |
| S45 | -6.287 | -2.78 | -3.21 | 1.04 | -0.05 | -5.65 | 0 | 26.93 | -21.86 |
| S46 | -6.286 | -2.88 | 40.73 | 3.03 | -1.44 | -7.59 | -1.89 | -7.64 | -28.07 |
| S47 | -6.245 | -3.84 | 34.41 | 0.57 | -1.59 | -2.16 | 0 | -18 | -17.07 |
| S48 | -6.174 | -3.94 | 24.61 | 2.04 | -0.87 | -6.06 | -1.72 | -4.37 | -17.57 |
| S49 | -6.168 | -3.94 | 24.61 | 2.04 | -0.87 | -6.06 | -1.72 | -4.37 | -17.57 |
| S50 | -6.152 | -4.07 | 27.78 | 0.68 | -0.26 | -5.47 | -2.34 | -2.05 | -22.41 |
| S51 | -6.141 | -5.68 | 44.25 | 2.07 | -2.28 | -10.39 | -1.4 | -13.06 | -24.87 |
| S52 | -6.115 | -5.68 | 44.25 | 2.07 | -2.28 | -10.39 | -1.4 | -13.07 | -24.87 |
| S53 | -6.113 | -8.04 | -16.05 | 7.25 | -1.54 | -3.03 | 0 | 27.9 | -22.57 |
| S54 | -6.109 | -8.18 | -16.05 | 7.25 | -1.54 | -3.03 | 0 | 27.76 | -22.56 |
| S55 | -6.08 | -8.68 | 38.32 | 5.15 | -1.25 | -9.96 | 0 | -16.57 | -24.38 |
| S56 | -6.03 | -9.31 | 40.54 | 1.33 | -1.25 | -8.8 | 0 | -16.89 | -24.24 |
| S57 | -6.016 | -10.44 | -8.54 | 1.92 | -0.19 | -9.28 | 0 | 29.97 | -24.33 |
| S58 | -5.908 | -10.64 | 31.22 | 0.83 | -1.18 | -3.9 | 0 | -13.93 | -23.67 |
| S59 | -5.906 | -12.08 | 54.33 | 1.93 | -1.58 | -12.36 | -1.03 | -27.51 | -25.86 |
| S60 | -5.906 | 14.17 | 24.44 | 2.54 | -1.84 | -4.98 | 0 | 10.02 | -16.01 |
| S61 | -5.886 | -14.75 | -9.17 | 0.52 | -1.29 | -7.32 | 0 | 25.75 | -23.25 |
| S62 | -5.886 | -14.55 | -9.16 | 0.52 | -1.29 | -7.31 | 0 | 25.94 | -23.26 |
| S63 | -5.886 | -13.97 | -15.51 | 1.09 | -1.54 | -2.32 | 0 | 19.3 | -14.99 |
| S64 | -5.872 | -14.81 | 13.88 | 4 | -0.87 | -8.64 | -1.03 | 1.29 | -23.43 |
| S65 | -5.82 | -15.45 | -13.08 | 1.08 | -1.26 | -3.1 | 0 | 18.05 | -17.15 |
| S66 | -5.711 | -15.65 | 31.86 | 0.54 | -1.33 | -11.67 | -1.19 | -10.97 | -22.88 |
| S67 | -5.688 | -16.16 | -16.73 | 3.44 | -1.76 | -3.45 | 0 | 23.3 | -20.95 |
| S68 | -5.683 | -16.8 | 32.19 | 0.72 | -0.94 | -10.97 | -1.19 | -13.2 | -23.42 |
| S69 | -5.676 | -16.83 | 40.19 | 0.54 | -0.79 | -10.78 | 0 | -21.38 | -24.61 |
| S70 | -5.67 | -17.01 | 42.65 | 0.13 | -1.33 | -12.64 | -0.89 | -22.26 | -22.66 |
| S71 | -5.661 | -17.04 | -9.22 | 0.24 | -0.57 | -6.36 | 0 | 22.02 | -23.16 |
| S72 | -5.656 | -17.3 | -22.06 | 4.53 | -1.59 | -8.46 | 0 | 27 | -16.73 |
| S73 | -5.656 | -17.06 | -7.29 | 0.08 | -0.29 | -9.13 | -0.99 | 26.15 | -25.6 |
| S74 | -5.63 | -17.97 | 27.43 | 3.25 | -1.6 | -13.35 | -1.02 | -9.5 | -23.18 |
| S75 | -5.62 | -18.01 | 27.43 | 3.25 | -1.6 | -13.35 | -1.02 | -9.54 | -23.18 |
| S76 | -5.618 | -18.12 | 30.9 | -0.3 | -1.46 | -11.33 | -1.29 | -11.38 | -23.26 |
| S77 | -5.588 | -18.21 | 16.05 | 1.17 | -0.86 | -5.96 | -2.26 | -7.6 | -18.74 |
| S78 | -5.588 | -18.18 | -9.29 | 0.97 | -1.21 | -11.22 | 0 | 22.59 | -20.01 |
| S79 | -5.565 | -18.22 | 30.86 | -0.3 | -1.46 | -11.32 | -1.29 | -11.48 | -23.23 |
| S80 | -5.544 | -18.26 | -9.29 | 0.97 | -1.21 | -11.22 | 0 | 22.51 | -20.02 |
| S81 | -5.539 | -18.27 | -15.96 | 2.18 | -1.39 | -5.26 | 0 | 22.69 | -20.52 |
| S82 | -5.525 | -18.29 | -17.39 | 0.46 | -1.23 | -3.51 | 0 | 18.82 | -15.44 |
| S83 | -5.503 | -18.77 | -10.41 | 0.7 | -0.49 | -7.13 | -3.34 | 19.36 | -17.47 |
| S84 | -5.491 | -19.45 | -4.7 | 1.53 | 0 | -11 | 0 | 21.72 | -27.01 |
| S85 | -5.487 | -19.95 | -9.7 | 1.73 | -0.39 | -14.07 | -1.02 | 29.49 | -26 |
| S86 | -5.465 | -20.08 | -13.03 | -0.13 | -1.01 | -5.46 | 0 | 19.48 | -19.93 |
| S87 | -5.462 | -20.13 | 33.96 | 0.54 | -1.34 | -12.99 | -1.41 | -10.73 | -28.14 |
| S88 | -5.434 | -20.18 | -9.78 | 1.75 | -0.39 | -14.06 | -1.02 | 29.31 | -25.99 |
| S89 | -5.423 | -20.5 | -21.05 | 0.86 | -0.59 | -9.46 | -1.18 | 34.26 | -23.34 |
| S90 | -5.391 | -20.72 | 28.58 | 2.31 | -2 | -11.4 | -1.29 | -10.67 | -26.25 |
| S91 | -5.357 | -20.75 | 28.58 | 2.31 | -2 | -11.4 | -1.29 | -10.7 | -26.25 |
| S92 | -5.348 | -20.86 | -13.73 | 0.85 | -0.46 | -8 | 0 | 26.02 | -25.54 |
| S93 | -5.254 | -21.42 | -16.89 | 0.78 | -0.52 | -10.97 | 0 | 30.6 | -24.43 |
| S94 | -5.243 | -21.62 | -11.22 | 0.64 | -0.52 | -8.16 | -2.12 | 17.73 | -17.97 |
| S95 | -5.243 | -21.45 | -7.8 | 1.47 | -1.83 | -5.37 | 0 | 16.43 | -24.36 |
| S96 | -5.234 | -21.69 | -10.82 | 2.04 | -0.52 | -10.61 | 0 | 21.41 | -23.2 |
| S97 | -5.228 | -22.1 | 34.79 | 2.12 | -1.56 | -11.36 | -1.28 | -17.56 | -27.24 |
| S98 | -5.196 | -22.22 | 29.13 | 3.07 | -1.5 | -13.38 | -0.95 | -13.67 | -24.92 |
| S99 | -5.124 | -22.23 | 34.79 | 2.09 | -1.55 | -11.37 | -1.28 | -17.71 | -27.19 |
| S100 | -5.092 | -22.6 | -10.43 | 4.71 | -1.32 | -10.11 | -1.32 | 20.79 | -24.92 |
| S101 | -5.089 | -22.61 | -12.96 | 0.96 | -1.21 | -7.21 | -1.85 | 22.82 | -23.18 |
| S102 | -5.037 | -22.61 | -12.96 | 0.96 | -1.21 | -7.21 | -1.85 | 22.82 | -23.18 |
| S103 | -5.007 | -22.67 | -14.22 | 1.53 | -1.3 | -7.99 | 0 | 23.51 | -24.21 |
| S104 | -4.985 | -22.73 | 32.78 | 1.02 | -0.85 | -14.54 | -0.97 | -12.15 | -28.03 |
| S105 | -4.918 | -23.49 | -16.06 | 0.52 | -1.57 | -3.11 | 0 | 16.92 | -20.17 |
| S106 | -4.887 | -23.5 | 33.42 | 0.46 | -1.26 | -11.55 | -1.4 | -13.51 | -29.66 |
| S107 | -4.818 | -23.62 | -17.17 | 2.84 | -1.79 | -6.74 | -1.69 | 20.23 | -19.31 |
| S108 | -4.813 | -23.65 | 31.75 | 0.85 | -1.41 | -11.72 | -1.43 | -14.23 | -27.46 |
| S109 | -4.786 | -23.8 | 35.21 | 0.5 | -1.99 | -13.25 | -1.05 | -22.1 | -21.12 |
| S110 | -4.748 | -23.81 | -12.4 | 1.22 | -1.11 | -5.14 | -2.36 | 19.12 | -23.14 |
| S111 | -4.743 | -24.21 | -19.29 | 1.22 | -1.12 | -8.79 | 0 | 30.04 | -26.26 |
| S112 | -4.692 | -24.77 | -20.35 | 1.99 | -1.11 | -6.9 | 0 | 21.19 | -19.59 |
| S113 | -4.692 | -24.6 | 30.44 | 0.19 | -1.9 | -11.4 | -1.64 | -15.46 | -24.83 |
| S114 | -4.664 | -24.83 | -11.61 | 3.12 | -1.26 | -5.21 | 0.00 | 12.29 | -22.17 |
| S115 | -4.638 | -25.06 | -14.34 | 0.51 | 0.00 | -14.39 | -1.59 | 29.42 | -24.66 |
| S116 | -4.586 | 13.63 | 21.78 | 3.76 | -0.58 | -3.54 | 0.00 | 21.58 | -29.37 |
| S117 | -4.502 | -25.37 | -14.56 | 1.61 | -1.7 | -6.82 | 0.00 | 17.48 | -21.37 |
| S118 | -4.5 | -25.39 | -14.57 | 1.61 | -1.7 | -6.82 | 0.00 | 17.47 | -21.37 |
| S119 | -4.428 | -25.86 | -14.36 | 1.45 | -1.31 | -10.84 | -1.34 | 24.38 | -23.84 |
| S120 | -4.402 | -26.04 | -8.47 | 2.21 | -1.16 | -6.27 | 0.00 | 13.19 | -25.54 |
| S121 | -4.382 | -26.12 | -15.54 | 1.82 | -1.38 | -9.21 | -1.76 | 26.88 | -26.93 |
| S122 | -4.377 | -27.17 | -18.29 | -0.26 | -0.77 | -12.47 | -2.08 | 27.14 | -20.43 |
| S123 | -4.368 | -27.41 | -11.42 | 0.92 | -1.16 | -9.47 | -1.84 | 22.47 | -26.91 |
| S124 | -4.299 | -27.56 | -14.94 | 0.19 | -0.55 | -13.54 | -1.4 | 32.15 | -29.47 |
| S125 | -4.299 | 15.57 | 48.54 | 2.51 | -1.03 | -1.94 | 0.00 | -9.36 | -23.14 |
| S126 | -4.24 | 13.43 | 29.68 | 2.39 | -1.54 | -4.85 | 0.00 | 5.36 | -17.61 |
| S127 | -4.238 | -29.49 | -7.18 | 0.45 | -1.23 | -11.49 | -1.1 | 19.79 | -28.74 |
| S128 | -4.238 | -29.26 | -8.78 | 0.81 | -1.39 | -10.78 | -1.15 | 20.26 | -28.22 |
| S129 | -4.226 | -29.6 | 38.81 | 1.22 | -1.82 | -13.27 | -0.98 | -26.69 | -26.86 |
| S130 | -4.191 | -29.7 | -41.55 | 0.97 | -0.73 | -7.42 | 0.00 | 40.25 | -21.22 |
| S131 | -3.941 | -29.93 | -18.77 | 0.64 | -1.49 | -10.08 | -1.41 | 22.41 | -21.23 |
| S132 | -3.854 | -30.74 | -13.39 | 3.47 | -1.69 | -10.95 | -1.51 | 19.52 | -26.2 |
| S133 | -3.815 | -30.74 | -13.39 | 3.47 | -1.69 | -10.95 | -1.51 | 19.52 | -26.2 |
| S134 | -3.815 | -30.74 | -13.39 | 3.47 | -1.69 | -10.95 | -1.51 | 19.52 | -26.2 |
| S135 | -3.757 | -31.41 | -15.22 | -0.46 | -1.3 | -11.52 | 0 | 20.62 | -23.51 |
| S136 | -3.693 | -31.45 | -15.22 | -0.47 | -1.3 | -11.53 | 0 | 20.58 | -23.51 |
| S137 | -3.692 | -31.55 | -15.22 | -0.46 | -1.3 | -11.53 | 0 | 20.48 | -23.51 |
| S138 | -3.683 | -31.72 | -15.2 | -0.48 | -1.3 | -11.53 | 0 | 20.31 | -23.52 |
| S139 | -3.683 | 19.4 | 41.68 | 0.14 | -0.7 | -3.89 | 0 | -1.15 | -16.68 |
| S140 | -3.569 | -33.55 | -24.65 | 3.58 | -1.79 | -11.84 | -2.35 | 27.16 | -23.67 |
| S141 | -3.569 | 33.43 | 82.46 | 0.46 | -1.09 | -0.97 | 0 | -17.52 | -29.91 |
| S142 | -2.958 | -36.27 | -9.69 | 0.36 | -1.06 | -12.14 | -0.95 | 12.5 | -25.29 |
| S143 | -2.958 | -34.14 | -30.25 | 1.23 | -0.63 | -15.36 | -1.04 | 35.76 | -23.86 |
| S144 | -2.943 | -39.37 | -44.37 | 1.48 | -1.49 | -6.49 | -2.22 | 34.06 | -20.34 |
| S145 | -2.857 | -40.97 | -47.3 | 2.63 | -1.63 | -8.11 | -2.22 | 35.13 | -19.47 |
| S146 | -2.857 | -40.04 | -53.34 | 1.25 | -1.63 | -11.59 | -1.1 | 53.11 | -26.76 |
| S147 | -2.715 | -43.12 | -46.28 | 2.78 | -1.65 | -8.99 | -2.04 | 29.72 | -16.65 |
| S148 | -2.713 | 17.33 | 46.61 | 3.18 | -1.28 | -1.32 | 0 | -15.65 | -14.22 |
| S149 | -6.57 | -33.62 | -59.35 | 0.53 | -1.89 | -2.43 | -1.37 | 48.64 | -17.75 |
| S150 | -6.54 | -29.95 | -23.67 | 5.01 | -2.45 | -9.81 | -3.23 | 30.17 | -25.97 |
| S151 | -6.48 | -29.66 | -31.22 | 3.02 | -2.27 | -10.34 | -3.32 | 38.65 | -24.18 |
| S152 | -6.46 | -29.41 | -12.86 | 2.08 | -2.8 | -9.51 | -3.25 | 24.12 | -27.2 |
| S153 | -6.43 | -28.45 | -21.36 | 0.9 | -1.34 | -9.86 | -3.35 | 33.59 | -27.02 |
| S154 | -6.35 | -26.6 | -13.58 | 1.01 | -1.89 | -11.55 | -0.94 | 24.85 | -24.49 |
| S155 | -6.3 | -25.71 | -7.62 | 0.88 | -1.62 | -10.69 | -1.16 | 19.34 | -24.83 |
| S156 | -6.27 | -25.69 | -16.66 | 0.55 | -2.84 | -6.01 | -0.97 | 27.97 | -27.73 |
| S157 | -6.23 | -25.54 | -10.17 | 2.75 | -1.24 | -7.49 | -3.28 | 24.13 | -30.25 |
| S158 | -6.22 | -25.17 | -24.44 | 5.31 | -2.45 | -7.5 | -2.28 | 29.66 | -23.49 |
| S159 | -6.18 | -24.94 | -13.64 | 1.03 | -1.89 | -6.04 | -0.96 | 23.12 | -26.57 |
| S160 | -6.17 | -24.44 | -15.58 | 0.91 | -1.88 | -9.14 | -0.94 | 27.97 | -25.78 |
| S161 | -6.13 | -24.43 | -8.57 | 1.49 | -2.22 | -8.05 | -2.5 | 22.07 | -26.67 |
| S162 | -6.13 | -24.36 | -17.38 | 0.42 | -2.27 | -9.16 | -0.95 | 31.51 | -26.53 |
| S163 | -6.1 | -23.91 | -13.22 | 0.89 | -1.98 | -9.81 | -0.97 | 26.07 | -24.9 |
| S164 | -5.98 | -35.52 | -46.71 | 2.46 | -2.06 | -10.95 | -1.07 | 48.86 | -26.05 |
| S165 | -5.95 | -23.9 | -19.44 | 2.61 | -1.78 | -10.8 | -3.17 | 34.55 | -25.86 |
| S166 | -5.9 | -23.79 | -15.4 | 1.01 | -2.04 | -6.01 | -0.97 | 25.62 | -26.01 |
| S167 | -5.83 | -22.7 | -11.91 | -1.96 | -1.99 | -9 | -1.19 | 26.7 | -23.34 |
| S168 | -5.81 | -22.23 | -10.07 | -1.97 | -1.95 | -7.73 | -1.16 | 23.77 | -23.11 |
| S169 | -5.81 | -21.92 | -23.61 | 4.19 | -1.75 | -9.15 | -2.81 | 37.94 | -26.75 |
| S170 | -5.68 | -19.98 | -16.25 | 0.45 | -2.01 | -4.77 | -1.16 | 27.56 | -23.8 |
| S171 | -5.63 | -19.7 | -17.88 | 1.54 | -0.8 | -5.16 | -0.97 | 26.79 | -23.23 |
| S172 | -5.56 | -17.79 | -22.5 | 3.26 | -1.2 | -6.43 | -3.12 | 35.19 | -23.01 |
| S173 | -5.4 | -17 | -14.77 | 1.54 | -0.75 | -7.33 | -3.03 | 30.02 | -22.69 |
| S174 | -5.29 | -16.23 | -17.37 | 0.34 | -0.48 | -3.64 | -0.95 | 30.48 | -24.62 |
| S175 | -5.28 | -14.91 | -13.9 | 3.92 | -0.52 | -5.1 | -1.02 | 24.55 | -22.84 |
| S176 | -5.14 | -14.56 | -18.91 | 2.74 | -1.39 | -10.37 | -1.05 | 37.78 | -23.37 |
| S177 | -5.07 | -13.48 | -5 | 2.01 | -1.64 | -5.63 | -2.06 | 18.86 | -20.01 |
| S178 | -5.04 | -12.27 | -21.46 | 0.64 | -1.84 | -1.12 | -1.93 | 33.43 | -19.98 |
| S179 | -5.02 | -11.75 | -8.13 | 1.25 | -1.66 | -4.08 | -2.44 | 22.4 | -19.08 |
| S180 | -5.01 | -11.2 | -9.64 | 1.9 | -1.6 | -4.47 | -2.33 | 24.05 | -19.1 |
| S181 | -4.91 | -11.16 | -8.71 | 2.3 | -1.63 | -4.68 | -2.17 | 23.37 | -19.63 |
| S182 | -4.87 | -5.19 | 52.08 | 0.71 | -1.31 | -7.73 | -1.06 | -24.47 | -23.4 |
| S183 | -4.82 | -2.81 | 57.97 | 2.16 | -0.77 | -3.6 | -1.1 | -34.88 | -22.59 |
| S184 | -4.75 | -2.78 | 56.68 | 0.58 | -0.79 | -1.53 | -0.92 | -31.99 | -24.81 |
| S185 | -4.51 | -1.06 | 45.25 | 0.45 | -1.32 | 0 | -0.98 | -20.41 | -24.04 |
| S186 | -4.3 | 32.69 | 115.93 | 0.6 | -1.88 | -1.24 | 0 | -57.48 | -23.24 |

**Table S2. Smiles representation of various proposed inhibitors of human CAII**

| S1 | O[S](=O)C1=CC=CC=C1Cl | S94 | O=C(O)[C@H]1N=c2ccccc2=C1Cl |
| --- | --- | --- | --- |
| S2 | O=[S@](O)c1cccc(Cl)c1F | S95 | C[C@@H](O)CNC(=O)CBr |
| S3 | O[S](=O)C1=CC(=CC=C1F)Cl | S96 | O[C@H](CCl)C[n+]1ccccc1 |
| S4 | O[S](=O)C1=CC=C(Cl)C=C1F | S97 | N[C@H](CC(=O)O)c1ccc(Cl)cc1 |
| S5 | O[S](=O)C1=CC=C(Cl)C=C1 | S98 | N[C@H](Cc1ccccc1Cl)C(=O)O |
| S6 | N[S](=O)(=O)CCC1=NC2=C([NH]1)C=CC=C2 | S99 | N[C@@H](CC(=O)O)c1ccc(Cl)cc1 |
| S7 | NO[S](=O)NCC1=NC=CC=C1 | S100 | COC(=O)[C@H](N)c1ccccc1 |
| S8 | NO[S](=O)NCC1=NN=CS1 | S101 | COC(=O)[C@H](N)c1cccc(F)c1 |
| S9 | N[S](=O)(=O)CC1=CC=CC=C1 | S102 | COC(=O)[C@H](N)c1cccs1 |
| S10 | NO[S](=O)NCC1=NC2=C([NH]1)C=CC=C2 | S103 | O=S1(=O)CC[C@@H](NCCCl)C1 |
| S11 | N[S](=O)(=O)CC1=NOC2=C1C=CC=C2 | S104 | CN1Cc2ccccc2C[C@H]1C(=O)O |
| S12 | CC(C([NH3+])C([O-])=O)C1=CC=CC=C1 | S105 | C/C(O)=N/[C@@H](C)CC=O |
| S13 | CCC(C)C1[NH2+]C(CS1)C([O-])=O | S106 | Cc1cc(Cl)ccc1[C@H](N)C(=O)O |
| S14 | CC1CNCCC1C(O)=O | S107 | NC(=O)c1ccc(Cl)cn1 |
| S15 | CN(C)C1=CC=C(C(=C1)C)[PH](O)=O | S108 | Cc1ccc([C@H](N)C(=O)O)c(Cl)c1 |
| S16 | OC(\C=C/C1=CC=CC=C1)[PH](O)=O | S109 | N[C@H](CC(=O)O)c1cccc(Cl)c1 |
| S17 | N[S](=O)(=O)NCC1=CC=C2OC(=O)C=CC2=C1 | S110 | CC(=O)Nc1ccoc1C |
| S18 | CN1CCC2=CC=CC=C2C(N)C1=O | S111 | O=C(CCl)NC1CCNCC1 |
| S19 | OC(=O)CC(C#N)C1=CC=CC=C1 | S112 | C[C@@H](Cl)C(=O)NCCO |
| S20 | NC1=CC(=C(F)C=C1)N2CCCC2=O | S113 | Cc1cc(Cl)cc([C@H](N)C(=O)O)c1 |
| S21 | O=[S@](O)c1ccc(Cl)cc1 | S114 | COP(=O)(C#CCl)OC |
| S22 | CC[C@@H](O)[C@H](Cl)C(=O)O | S115 | C[N+](C)(C)CCc1ccc(Cl)cc1 |
| S23 | CC[C@@H](C)P(=O)(O)O | S116 | O=C(CCl)C1=C(O)CCCC1=O |
| S24 | CC[C@H](C)P(=O)(O)O | S117 | C[C@H](Cl)C(=O)NC[C@@H](C)O |
| S25 | CC(C)(C)P(=O)(O)O | S118 | C[C@H](O)CNC(=O)[C@H](C)Cl |
| S26 | CC(C)(Nc1ccccc1)C(=O)O | S119 | NNC(=O)Cc1ccc(Cl)cc1 |
| S27 | OC(=S)C[C@@H](O)C(O)=S | S120 | CC(=O)CCC1C(=O)CCC1=O |
| S28 | OC(=S)C[C@H](O)C(O)=S | S121 | NNC(=O)Cc1cccc(Cl)c1 |
| S29 | CCP(=O)(O)O | S122 | COc1ccc([C@H](C)N)cc1Cl |
| S30 | O=C(O)C1CC(O)C1 | S123 | COC(=O)[C@H](N)c1cccc(Cl)c1 |
| S31 | COC[C@@H](Br)C(=O)O | S124 | Cc1cc(Cl)ccc1NC=[N+](C)C |
| S32 | COC[C@H](Br)C(=O)O | S125 | C[C@H](Br)C(=O)C(=O)O |
| S33 | Nc1ccc(CC(=O)O)cc1 | S126 | CC[C@H](O)[C@@H](Cl)C(=O)O |
| S34 | N[C@H](C(=O)O)c1ccccc1S | S127 | C[C@@H](Cl)C(=O)NCc1ccncc1 |
| S35 | C[C@H](C(=O)O)N1CCc2ccccc21 | S128 | O=C(CCl)NCc1ccccn1 |
| S36 | O=C(O)CNc1ccc(F)cc1 | S129 | N[C@H](CC(=O)O)c1ccccc1Cl |
| S37 | Cc1ncc(CO)c(CO)c1S | S130 | Cc1nc(CCl)n(C)n1 |
| S38 | C[C@@H](C(=O)O)N1CCc2ccccc21 | S131 | N#C[C@@H](N)c1ccc(Cl)cc1 |
| S39 | Cc1ccccc1NC(C)(C)C(=O)O | S132 | COC(=O)[C@@H](N)c1cccc(Cl)c1 |
| S40 | CO/C(=C\C(=O)O)[C@H](C)Cl | S133 | COC(=O)[C@@H](N)c1ccc(Cl)cc1 |
| S41 | CO/C(=C\C(=O)O)[C@@H](C)Cl | S134 | COC(=O)[C@H](N)c1ccc(Cl)cc1 |
| S42 | CO/C(=C/C(=O)O)[C@H](C)Cl | S135 | CN[C@@H]1[C@H](Cl)CN2CC[C@@H](O)[C@@H]12 |
| S43 | Cc1ccc([P@@H](=O)O)cc1 | S136 | CN[C@@H]1[C@@H](Cl)CN2CC[C@@H](O)[C@@H]12 |
| S44 | CO/C(=C/C(=O)O)[C@@H](C)Cl | S137 | CN[C@H]1[C@@H](Cl)CN2CC[C@@H](O)[C@@H]12 |
| S45 | CC(C)C(=O)NC(=O)CCl | S138 | CN[C@H]1[C@@H](Cl)CN2CC[C@@H](O)[C@H]12 |
| S46 | C[S@@](=O)c1cccc(CC(=O)O)c1 | S139 | COC[C@H](Cl)C(=O)O |
| S47 | C[C@@H](S)[C@H](N)C(=O)O | S140 | N#Cc1cc(Cl)ccc1[C@H](N)CO |
| S48 | O=[P@@H](O)c1ccccc1O | S141 | C[C@@H](NC(=S)S)C(=O)O |
| S49 | O=[P@@H](O)[C@@H](O)c1ccccc1 | S142 | CN(C(=O)CC#N)c1ccccc1 |
| S50 | O=[P@@H](O)C1Cc2ccccc2C1 | S143 | CC[N+](C)(N)Cc1ccccc1Cl |
| S51 | O=C(O)[C@H]1Cc2cc(Cl)ccc2N1 | S144 | O=[S@@](O)c1cccc(Cl)c1 |
| S52 | O=C(O)[C@@H]1Cc2cc(Cl)ccc2N1 | S145 | O=[S@](O)c1ccc(F)cc1Cl |
| S53 | C[C@@]1(C(=O)NN)C[C@@H]1Br | S146 | N/C(=N\C(=O)CCl)c1ccccc1 |
| S54 | C[C@@]1(C(=O)NN)C[C@H]1Br | S147 | O=[S@](O)c1ccc(Cl)cc1 |
| S55 | CC1CCN([C@H](C(=O)O)C(C)C)CC1 | S148 | C[C@@](N)(F)C(=O)O |
| S56 | CC[C@H](C(=O)O)N1CCC[C@H](C)C1 | S149 | NO[S](=O)NCC1=CC=CS1 |
| S57 | CN1C[C@H]([N+](C)(C)C)CCCCC1=O | S150 | N[S](=O)(=O)CC1=CC=C2OC(=O)C=CC2=C1 |
| S58 | N[C@H](C/C=C/Br)C(=O)O | S151 | N[S](=O)(=O)NC1=CC=C2OC(=O)C=CC2=C1 |
| S59 | C[C@@H](CC(=O)O)c1ccccc1F | S152 | N[S](=O)(=O)CCC1=NOC2=C1C=CC=C2 |
| S60 | CCCCP(=O)(O)O | S153 | N[S](=O)(=O)CCC1=CC=C2OC(=O)C=CC2=C1 |
| S61 | C[C@]1(C(=O)NN)CC1(Cl)Cl | S154 | N[S](=O)(=O)CCC1=CC=CC=C1 |
| S62 | C[C@@]1(C(=O)NN)CC1(Cl)Cl | S155 | N[S](=O)(=O)CCC1=CC=CS1 |
| S63 | C[C@@H](I)C(N)=O | S156 | N[S](=O)(=O)NCC1=NC=NC=N1 |
| S64 | O=[P@@H](O)CCc1ccncc1 | S157 | N[S](=O)(=O)C1=CC=C2OC(=O)C=CC2=C1 |
| S65 | O=C1CC[C@H](C(=O)Cl)N1 | S158 | N[S](=O)(=O)CC1=NC2=C([NH]1)C=CC=C2 |
| S66 | N[C@@H](C(=O)O)c1cccc(Cl)c1 | S159 | N[S](=O)(=O)CCC1=NN=CS1 |
| S67 | C/C(N)=C(\C#N)C(=O)CCl | S160 | N[S](=O)(=O)CCC1=CC=NC=C1 |
| S68 | N[C@@H](C(=O)O)c1ccccc1Cl | S161 | NO[S](=O)NC1=NOC2=CC=CC=C12 |
| S69 | O=C(O)CN[C@H]1C=CC=CC1 | S162 | N[S](=O)(=O)NCC1=CC=NC=C1 |
| S70 | C[C@@H](C(=O)O)[C@H](N)c1ccccc1 | S163 | N[S](=O)(=O)CCC1=NC=CC=C1 |
| S71 | CCN(CC)C(=O)C(C)=O | S164 | NO[S](=O)NCC1=CC=CC=C1 |
| S72 | CCS[C@@H]1CC[C@@]1(O)CN | S165 | N[S](=O)(=O)NC1=NC2=CC=CC=C2[NH]1 |
| S73 | CNC(=O)c1csnc1C | S166 | N[S](=O)(=O)CCC1=NC=NC=N1 |
| S74 | N[C@@H](Cc1cccc(Cl)c1)C(=O)O | S167 | N[S](=O)(=O)CC1=CC=CC=N1 |
| S75 | N[C@H](Cc1cccc(Cl)c1)C(=O)O | S168 | N[S](=O)(=O)CC1=CC=CS1 |
| S76 | N[C@@H](C(=O)O)c1ccc(Cl)cc1 | S169 | N[S](=O)(=O)NCC1=NOC2=C1C=CC=C2 |
| S77 | O=[P@@H](O)c1ccc(Cl)cc1 | S170 | N[S](=O)(=O)CC1=NC=NC=N1 |
| S78 | NCC1=C[C@H]2OCO[C@@H]2C=C1Cl | S171 | N[S](=O)(=O)CC1=NN=CS1 |
| S79 | N[C@H](C(=O)O)c1ccc(Cl)cc1 | S172 | N[S](=O)(=O)C1=NC2=C([NH]1)C=CC=C2 |
| S80 | NCC1=C[C@@H]2OCO[C@@H]2C=C1Cl | S173 | N[S](=O)(=O)C1=NOC2=CC=CC=C12 |
| S81 | CN(C(=O)[C@@H]1CSCN1)C1CC1 | S174 | N[S](=O)(=O)C1=NN=CS1 |
| S82 | NC(=O)CCCCl | S175 | N[S](=O)(=O)NC1=CC=CC=N1 |
| S83 | CC1=c2cc(F)ccc2=N[C@@H]1C(=O)O | S176 | N[S](=O)(=O)NC1=CC=CC=C1 |
| S84 | CC[C@H](CCl)[n+]1ccccc1 | S177 | N[S](=O)(=O)C1=CC=CC=C1 |
| S85 | COC(=O)[C@@H](N)c1ccccc1Cl | S178 | N[S](=O)(=O)C1=NC=NC=N1 |
| S86 | O=C(CCl)NC1CCOCC1 | S179 | N[S](=O)(=O)C1=CC=NC=C1 |
| S87 | Cc1ccc([C@H](N)C(=O)O)cc1Cl | S180 | N[S](=O)(=O)C1=CC=CS1 |
| S88 | COC(=O)[C@H](N)c1ccccc1Cl | S181 | N[S](=O)(=O)C1=NC=CC=C1 |
| S89 | C[C@@H](CCN)n1cc(Cl)cn1 | S182 | N[S](=O)(=O)NC1=CC=NC=C1 |
| S90 | C[C@@H](CCN)n1cc(Cl)cn1 | S183 | N[S](=O)(=O)NC1=CC=CS1 |
| S91 | N[C@H](Cc1ccc(Cl)cc1)C(=O)O | S184 | N[S](=O)(=O)NC1=NN=CS1 |
| S92 | O=C(CCCl)N1CCNCC1 | S185 | N[S](=O)(=O)NC1=NC=NC=N1 |
| S93 | Cc1cc[n+](C[C@H](O)CCl)cc1 | S186 | N[S](=O)(=O)CC1=CC=NC=C1 |

**Table S3. Different pharmacophore parametric scores for the generated hypothesis AADRR_3**

|  | **Activity** | **Fitness** | **Site score** | **Vector score** | **Volume** | **Matched ligand site** |
| --- | --- | --- | --- | --- | --- | --- |
| **AADRR_3** | active | 1.89 | 0.24 | 0.922 | 0.728 | A(2) A(3) D(5) R(7) R(8) |
|  | active | 2.141 | 0.482 | 0.866 | 0.793 | A(2) A(4) D(8) R(10) R(11) |
|  | active | 2.693 | 0.816 | 0.993 | 0.883 | A(3) A(2) D(6) R(7) R(8) |
|  | active | 3 | 1 | 1 | 1 | A(2) A(3) D(6) R(8) R(9) |
|  | active | 2.999 | 0.999 | 1 | 1 | A(2) A(3) D(5) R(8) R(9) |
|  | active | 2.993 | 0.995 | 1 | 0.998 | A(2) A(3) D(6) R(8) R(9) |
|  | active | 2.083 | 0.387 | 0.956 | 0.74 | A(2) A(3) D(5) R(7) R(8) |
|  | active | 1.878 | 0.421 | 0.728 | 0.728 | A(2) A(1) D(5) R(8) R(7) |
|  | active | 2.015 | 0.351 | 0.961 | 0.703 | A(1) A(2) D(5) R(8) R(7) |
|  | inactive | 1.496 | 0.372 | 0.684 | 0.53 | A(3) A(2) D(4) R(6) R(-) |
|  | inactive | 1.8 | 0.381 | 0.947 | 0.564 | A(2) A(3) D(5) R(7) R(-) |
|  | inactive | 1.89 | 0.417 | 0.98 | 0.597 | A(2) A(3) D(4) R(7) R(-) |
|  | inactive | 1.992 | 0.523 | 0.987 | 0.624 | A(2) A(3) D(5) R(7) R(-) |
|  | inactive | 1.492 | 0.365 | 0.686 | 0.529 | A(4) A(3) D(5) R(8) R(-) |
|  | inactive | 1.852 | 0.428 | 0.957 | 0.574 | A(3) A(4) D(5) R(8) R(-) |
|  | inactive | 1.99 | 1 | 1 | 0.622 | A(3) A(2) D(5) R(-) R(-) |
|  | inactive | 2.085 | 1 | 1 | 0.718 | A(3) A(2) D(5) R(-) R(-) |
|  | inactive | 1.854 | 0.441 | 0.88 | 0.644 | A(1) A(2) D(5) R(8) R(-) |
|  | inactive | 1.42 | 0.324 | 0.685 | 0.487 | A(4) A(3) D(6) R(7) R(-) |
|  | inactive | 1.42 | 0.325 | 0.684 | 0.487 | A(4) A(3) D(5) R(7) R(-) |
|  | inactive | 1.811 | 0.412 | 0.958 | 0.544 | A(4) A(3) D(5) R(7) R(-) |
|  | inactive | 1.784 | 0.393 | 0.949 | 0.539 | A(4) A(3) D(6) R(8) R(-) |
|  | inactive | 1.785 | 0.393 | 0.949 | 0.54 | A(4) A(3) D(5) R(8) R(-) |
|  | inactive | 1.814 | 0.474 | 0.847 | 0.617 | A(3) A(4) D(7) R(10) R(-) |

**Table S4. Different pharmacophore parametric scores for the generated hypothesis AADH_1**

|  | **Activity** | **Fitness** | **Site score** | **Vector score** | **Volume** | **Matched ligand site** |
| --- | --- | --- | --- | --- | --- | --- |
| **AADH_1** | active | 1.815 | 0.41 | 0.696 | 0.709 | A(2) A(1) D(4) H(5) |
|  | active | 1.788 | 0.427 | 0.69 | 0.672 | A(1) A(2) D(3) H(5) |
|  | active | 1.8 | 0.427 | 0.69 | 0.683 | A(2) A(1) D(4) H(5) |
|  | active | 1.81 | 0.41 | 0.696 | 0.704 | A(2) A(1) D(4) H(6) |
|  | active | 1.803 | 0.427 | 0.69 | 0.686 | A(2) A(1) D(4) H(5) |
|  | active | 3 | 1 | 1 | 1 | A(1) A(2) D(3) H(5) |
|  | active | 0.715 | -0.109 | 0.567 | 0.257 | A(1) A(2) D(3) H(5) |
|  | inactive | 1.896 | 0.782 | 0.984 | 0.447 | A(1) A(2) D(-) H(3) |
|  | inactive | 1.858 | 0.782 | 0.984 | 0.409 | A(2) A(1) D(-) H(3) |
|  | inactive | 1.863 | 0.739 | 0.988 | 0.424 | A(2) A(-) D(3) H(4) |
|  | inactive | 1.712 | 0.594 | 0.991 | 0.331 | A(2) A(1) D(-) H(3) |
|  | inactive | 1.121 | 0.313 | 0.604 | 0.295 | A(1) A(2) D(4) H(-) |
|  | inactive | 1.759 | 0.581 | 0.995 | 0.382 | A(2) A(-) D(3) H(5) |
|  | inactive | 1.546 | 0.38 | 0.582 | 0.698 | A(3) A(2) D(5) H(-) |
|  | inactive | 1.601 | 0.717 | 0.663 | 0.495 | A(-) A(1) D(5) H(6) |
|  | inactive | 1.471 | 0.243 | 0.744 | 0.552 | A(-) A(2) D(3) H(5) |
|  | inactive | 1.119 | 0.173 | 0.635 | 0.358 | A(2) A(-) D(3) H(5) |
|  | inactive | 1.388 | 0.428 | 0.653 | 0.439 | A(1) A(2) D(4) H(-) |
|  | inactive | 1.664 | 0.412 | 0.82 | 0.558 | A(1) A(2) D(-) H(4) |
|  | inactive | 1.859 | 0.534 | 0.756 | 0.747 | A(2) A(1) D(-) H(3) |
|  | inactive | 1.195 | 0.382 | 0.547 | 0.38 | A(1) A(-) D(2) H(3) |
|  | inactive | 1.061 | 0.341 | 0.551 | 0.269 | A(1) A(-) D(2) H(4) |
|  | inactive | 1.273 | 0.428 | 0.63 | 0.347 | A(1) A(3) D(4) H(-) |
|  | inactive | 1.306 | 0.428 | 0.63 | 0.381 | A(1) A(3) D(4) H(-) |
|  | inactive | 1.801 | 0.822 | 0.797 | 0.528 | A(2) A(1) D(-) H(6) |
|  | inactive | 1.631 | 0.304 | 0.778 | 0.55 | A(3) A(2) D(4) H(5) |
|  | inactive | 1.856 | 0.385 | 0.953 | 0.518 | A(3) A(2) D(5) H(7) |
|  | inactive | 1.682 | 0.532 | 0.692 | 0.458 | A(3) A(2) D(5) H(7) |
|  | inactive | 1.494 | 0.361 | 0.706 | 0.427 | A(3) A(1) D(4) H(5) |
|  | inactive | 1.306 | 0.371 | 0.527 | 0.408 | A(2) A(1) D(4) H(5) |
|  | inactive | 2.060 | 0.605 | 0.804 | 0.651 | A(3) A(2) D(6) H(7) |
|  | inactive | 2.004 | 0.605 | 0.804 | 0.595 | A(3) A(2) D(6) H(7) |
|  | inactive | 1.473 | 0.329 | 0.707 | 0.437 | A(3) A(1) D(4) H(5) |

**Table S5.** Evaluated toxicity parameters.

| **Target** | **S1** | **S2** | **S3** | **S6** | **S7** | **S8** | **S11** | **S12** | **S13** | **S14** | **S15** | **S16** |
| --- | --- | --- | --- | --- | --- | --- | --- | --- | --- | --- | --- | --- |
| Predicted LD_50_ (mg/kg) | 1190 | 500 | 500 | 49 | 1700 | 1000 | 1829 | 2400 | 1600 | 5490 | 1345 | 2500 |
| Predicted Toxicity Class | 4 | 4 | 4 | 2 | 2 | 4 | 4 | 5 | 4 | 6 | 4 | 5 |
| Hepatotoxicity | 0.69 | 0.80 | 0.80 | 0.63 | 0.61 | 0.58 | 0.54 | 0.62 | 0.65 | 0.81 | 0.65 | 0.72 |
| Carcinogenicity | 0.62 | 0.84 | 0.84 | 0.50 | 0.54 | 0.61 | 0.54 | 0.68 | 0.53 | 0.58 | 0.55 | 0.85 |
| Immunotoxicity | 0.96 | 0.98 | 0.95 | 0.96 | 0.93 | 0.97 | 0.52 | 0.62 | 0.57 | 0.51 | 0.63 | 0.58 |
| Mutagenicity | 0.97 | 0.88 | 0.88 | 0.59 | 0.50 | 0.50 | 0.88 | 0.56 | 0.55 | 0.59 | 0.55 | 0.60 |
| Cytotoxicity | 0.93 | 0.80 | 0.80 | 0.68 | 0.60 | 0.60 | 0.86 | 0.53 | 0.54 | 0.62 | 0.68 | 0.64 |
| Aryl hydrocarbon Receptor (AhR) | 0.97 | 0.95 | 0.95 | 0.75 | 0.76 | 0.78 | 0.91 | 0.97 | 0.96 | 0.98 | 0.59 | 0.94 |
| Androgen Receptor (AR) | 0.99 | 0.94 | 0.94 | 0.98 | 0.96 | 0.97 | 0.98 | 0.99 | 0.98 | 0.99 | 0.97 | 0.96 |
| Androgen Receptor Ligand Binding Domain(AR-LBD) | 0.99 | 0.98 | 0.98 | 0.99 | 0.97 | 0.98 | 0.99 | 0.98 | 0.98 | 0.99 | 0.93 | 0.95 |
| Aromatase | 1.00 | 0.91 | 0.91 | 0.87 | 0.95 | 0.97 | 0.96 | 0.98 | 0.97 | 0.97 | 0.94 | 0.98 |
| Estrogen Receptor Alpha(ER) | 0.99 | 0.94 | 0.94 | 0.93 | 0.80 | 0.82 | 0.94 | 0.91 | 0.89 | 0.94 | 0.64 | 0.90 |
| Estrogen Receptor Ligand Binding Domain(ER-LBD) | 1.0 | 0.96 | 0.96 | 0.97 | 0.94 | 0.94 | 0.98 | 0.98 | 0.96 | 0.98 | 0.99 | 0.91 |
| Peroxisome Proliferator-Activated receptor Gamma (PPAR Gamma) | 0.99 | 0.95 | 0.95 | 0.98 | 0.98 | 0.98 | 0.99 | 0.99 | 0.95 | 0.95 | 0.99 | 0.97 |
| Nuclear factor (erythroid-derived 2)-like2/antioxidant responsive element (nrf2/ARE) | 0.88 | 0.84 | 0.84 | 0.95 | 0.93 | 0.94 | 0.96 | 0.99 | 0.95 | 0.98 | 0.85 | 0.89 |
| Heat shock factor response element (HSE) | 0.88 | 0.84 | 0.84 | 0.95 | 0.93 | 0.94 | 0.96 | 0.99 | 0.95 | 0.98 | 0.85 | 0.89 |
| Mitochondrial Membrane Potential (MMP) | 0.70 | 0.88 | 0.88 | 0.92 | 0.89 | 0.90 | 0.95 | 0.98 | 0.93 | 0.95 | 0.87 | 0.92 |
| Phosphoprotein (Tumour Suppressor)p53 | 0.96 | 0.96 | 0.96 | 0.92 | 0.92 | 0.95 | 0.96 | 0.99 | 0.96 | 0.97 | 0.92 | 0.88 |
| ATPase family AAA domain containingprotein5(ATAD5) | 0.99 | 0.97 | 0.97 | 0.97 | 0.94 | 0.95 | 0.98 | 0.98 | 0.98 | 0.99 | 0.91 | 0.92 |

**Figure S1.** Pharmacophore model depicting the alignment of various active and inactive ligands along with the predicted features for various sulphonamide derived ligands in the dataset.

| **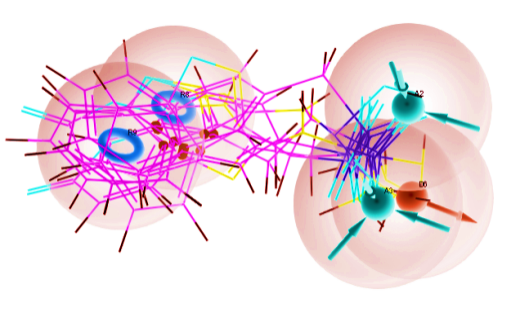** | **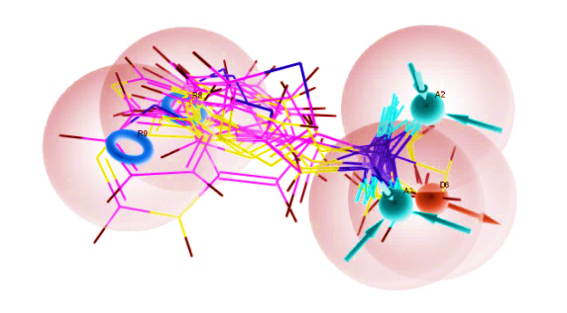** | **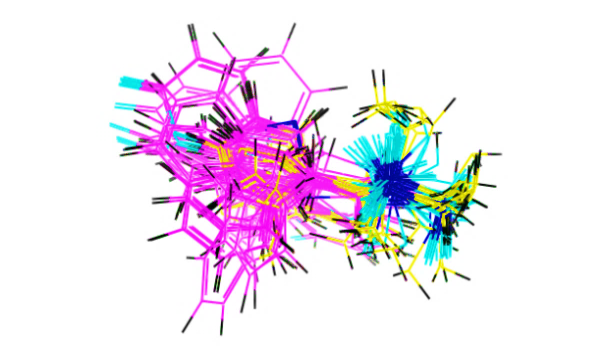** |
| --- | --- | --- |
| **Active** | **Inactive** | **Aligned** |

**Figure S2:** Pharmacophore model depicting the alignment of various active and inactive ligands along with the predicted features for various non-sulphonamide derived ligands in the dataset.

| 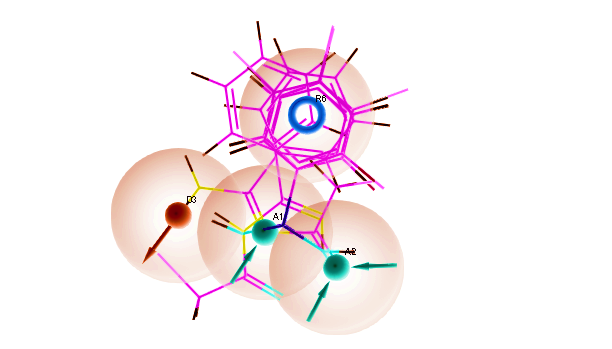 | **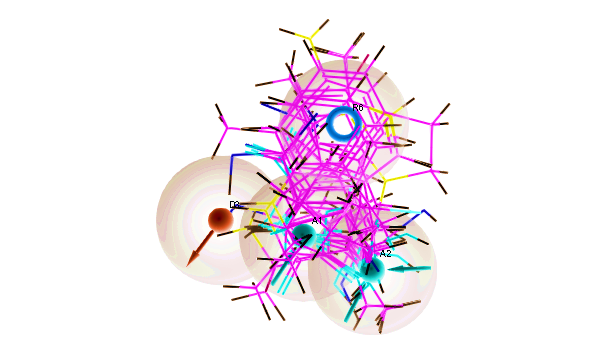** | **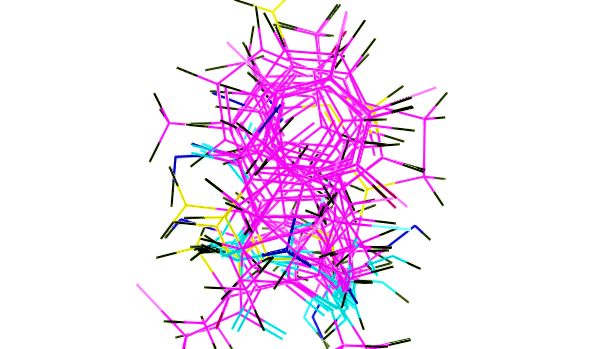** |
| --- | --- | --- |
| **Active** | **Inactive** | **Aligned** |

**Figure S3.** Docked poses of various proposed sulphonamide inhibitors against human CA(II)

| **S3** | **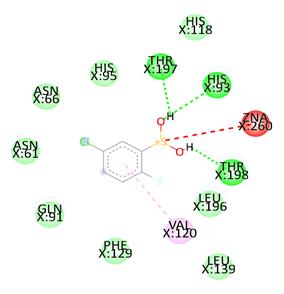** | **S4** | **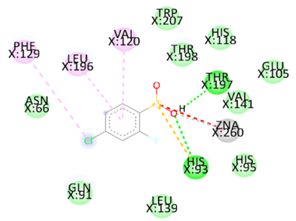** |
| --- | --- | --- | --- |
| **S5** | **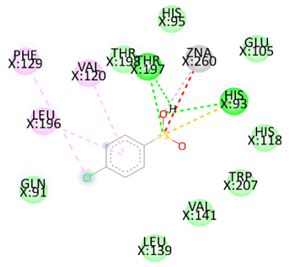** | **S9** | **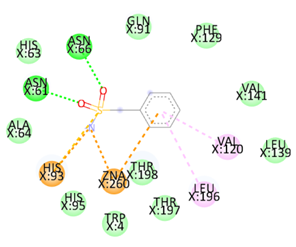** |
| **S10** | **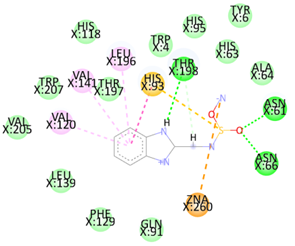** | **S11** | **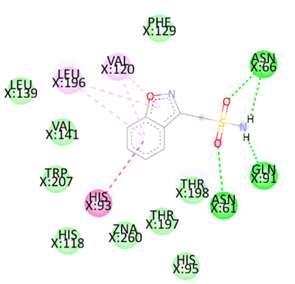** |
| **S12** | **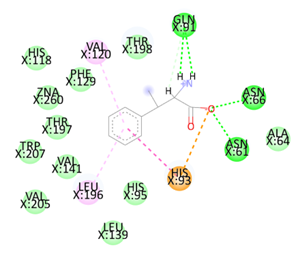** | **S13** | **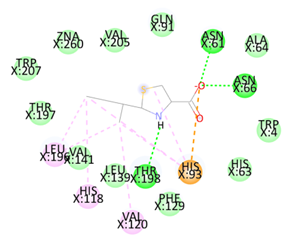** |
| **S14** | **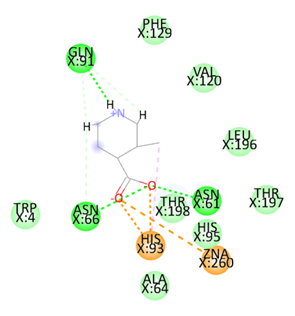** | **S17** | **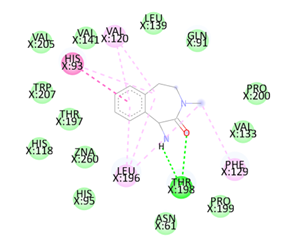** |
| **S18** | **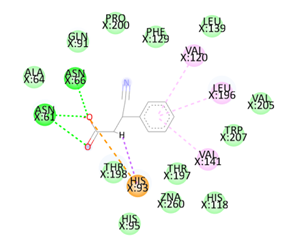** | **S19** | **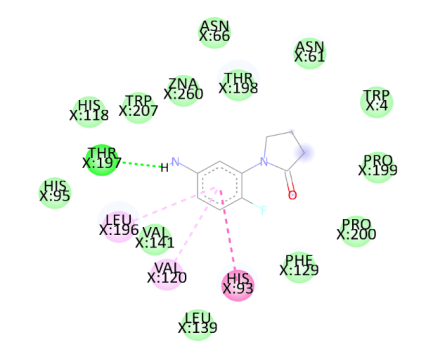** |
| **S20** | **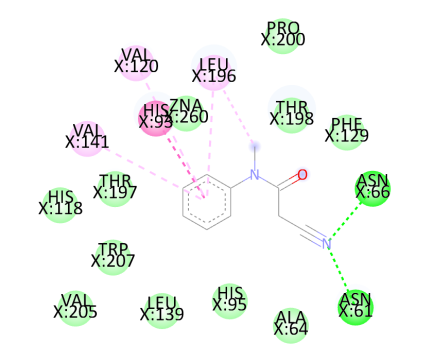** | **S21** | **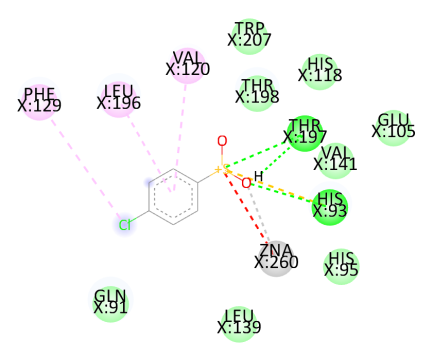** |
| **S22** | **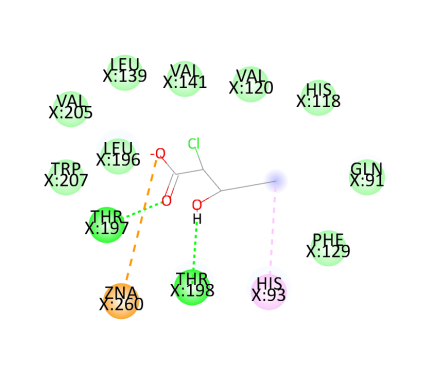** | **S23** | **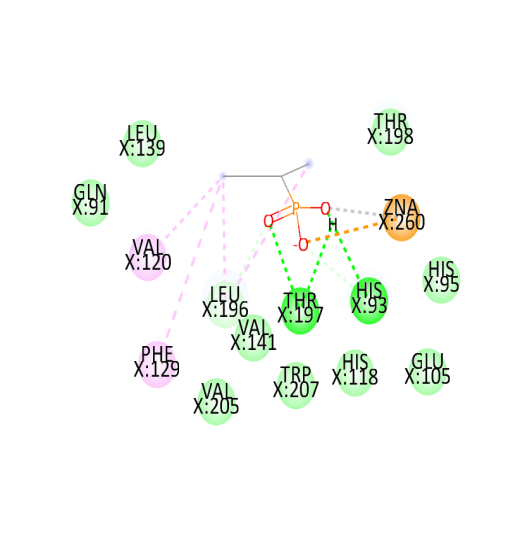** |
| **S24** | **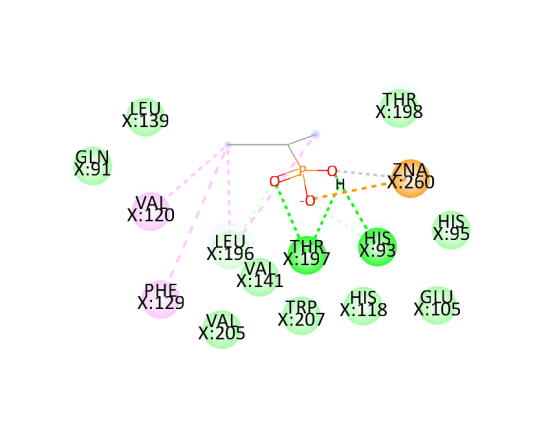** | **S25** | **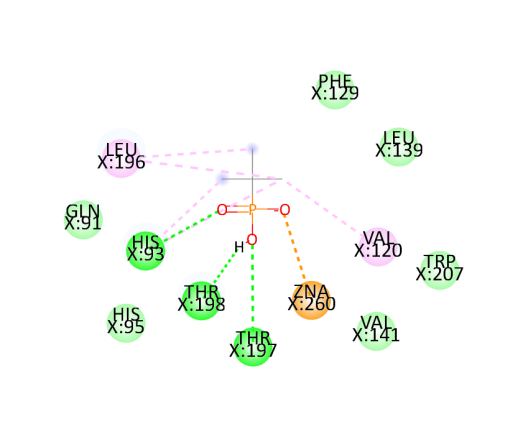** |
| **S26** | **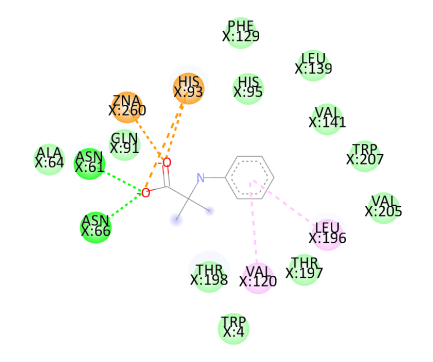** | **S27** | **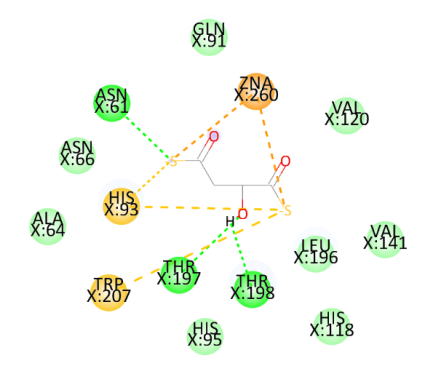** |
| **S28** | **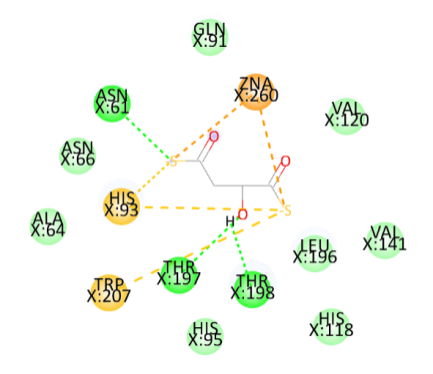** | **S29** | **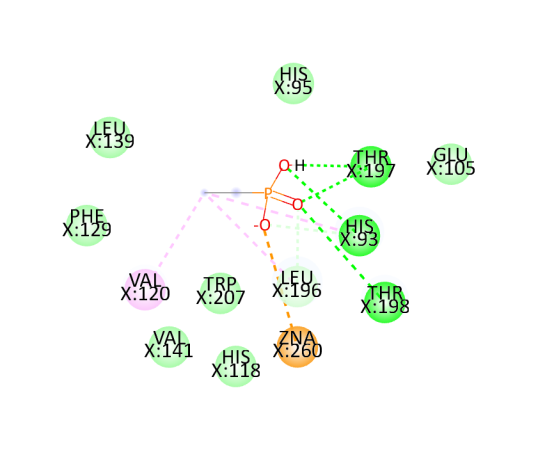** |
| **S30** | **** | **S31** | **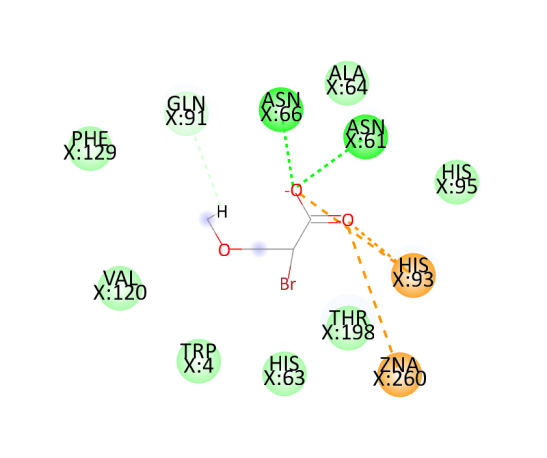** |
| **S32** | **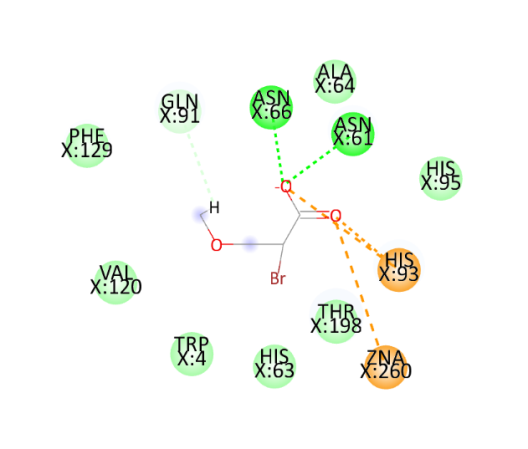** | **S33** | **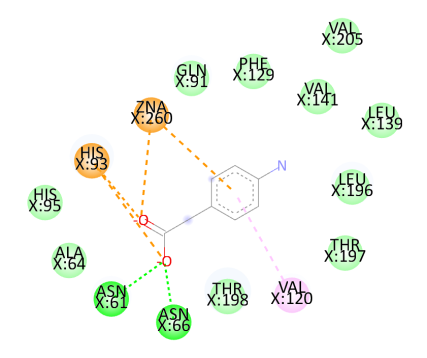** |
| **S34** | **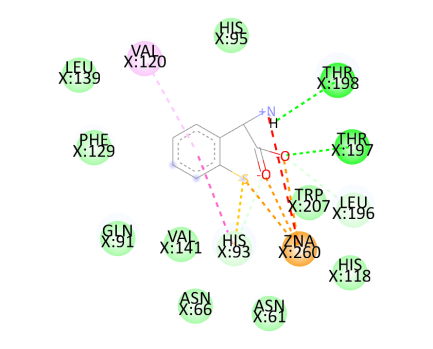** | **S35** | **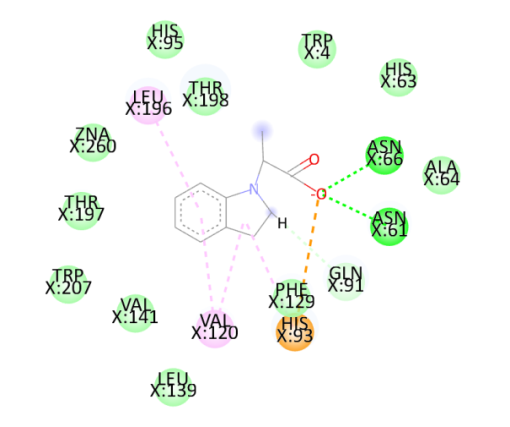** |
| **S36** | **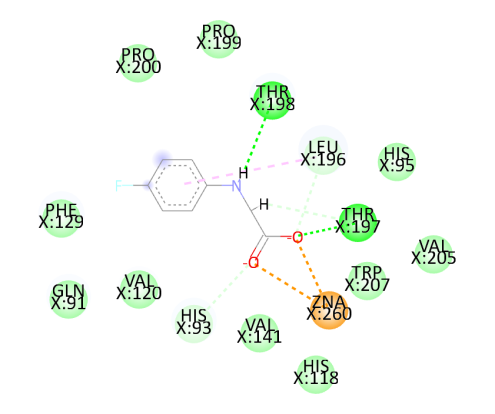** | **S37** | **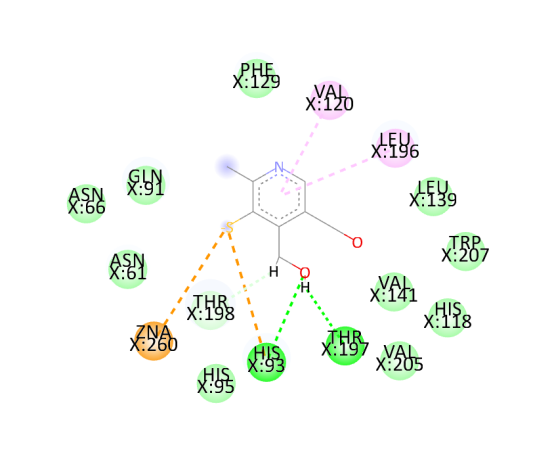** |
| **S38** | **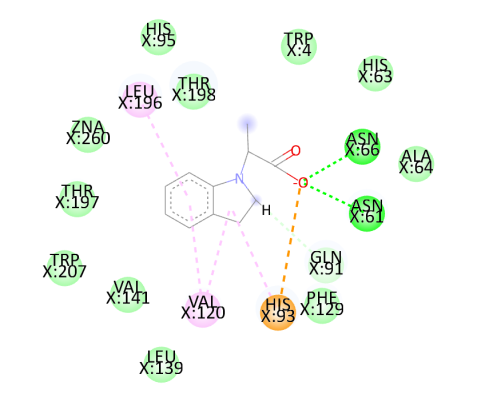** | **S39** | **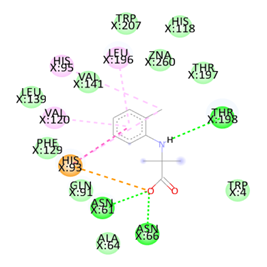** |
| **S40** | **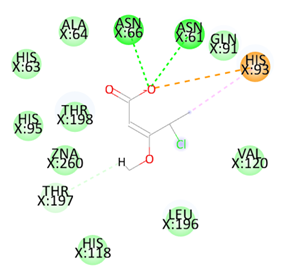** | **S41** | **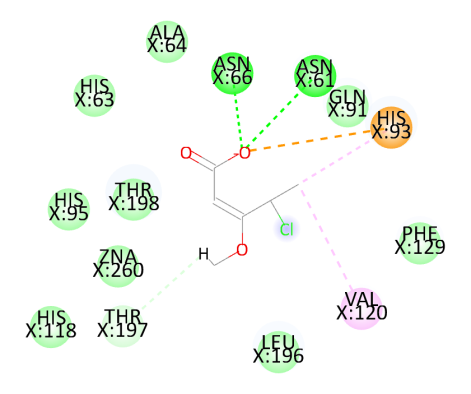** |
| **S42** | **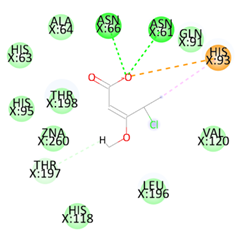** | **S43** | **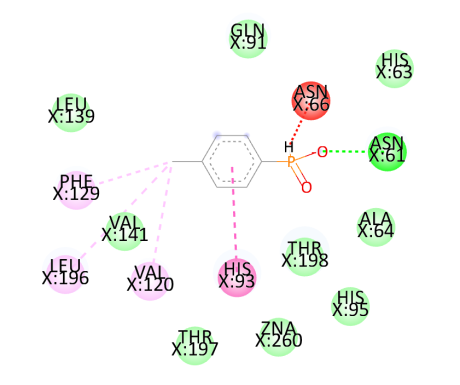** |
| **S44** | **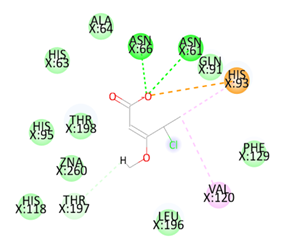** | **S45** | **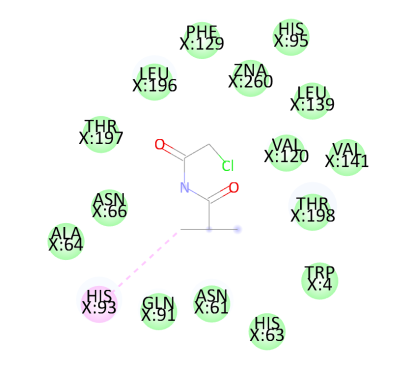** |
| **S46** | **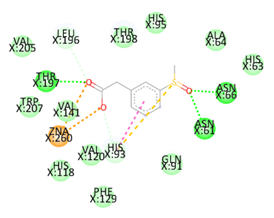** | **S47** | **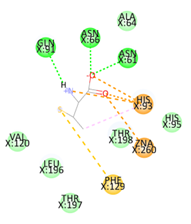** |
| **S48** | **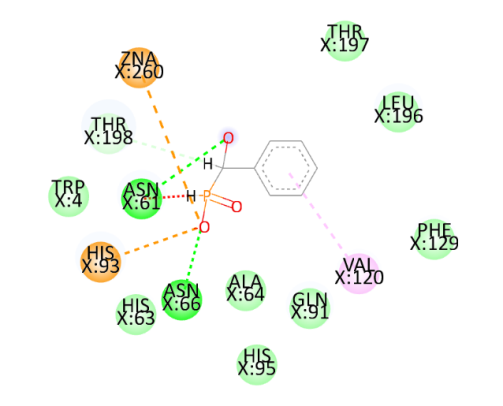** | **S49** | **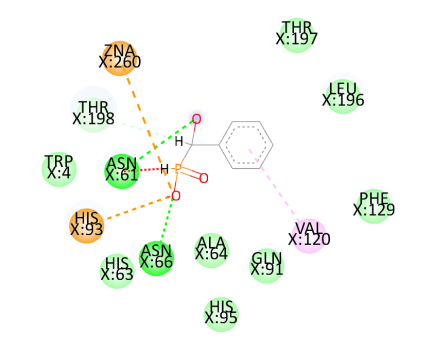** |
| **S50** | **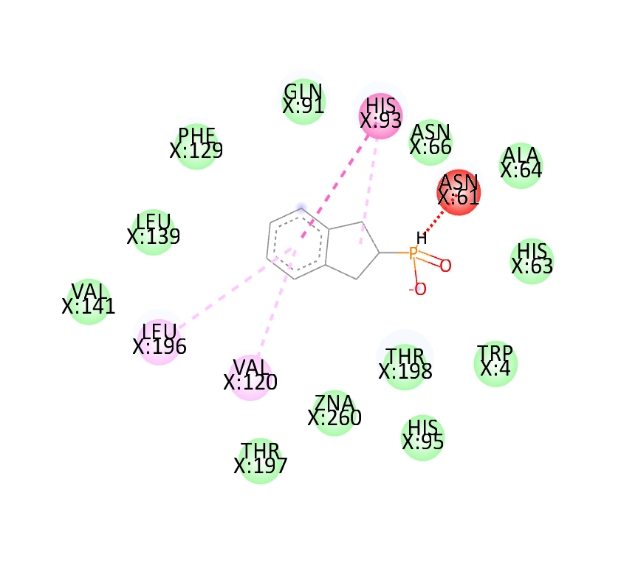** | **S51** | **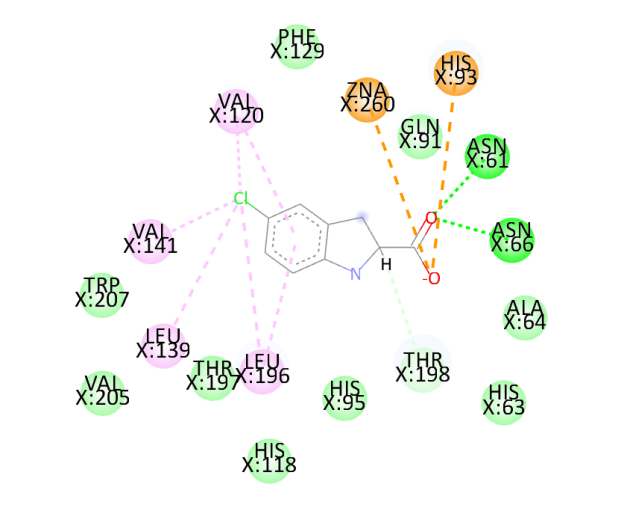** |
| **S52** | **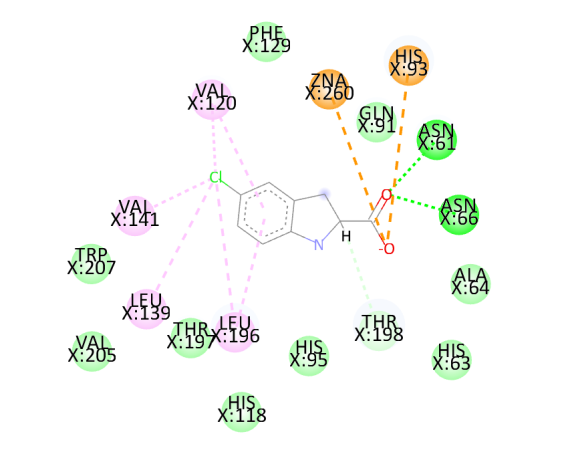** | **S53** | **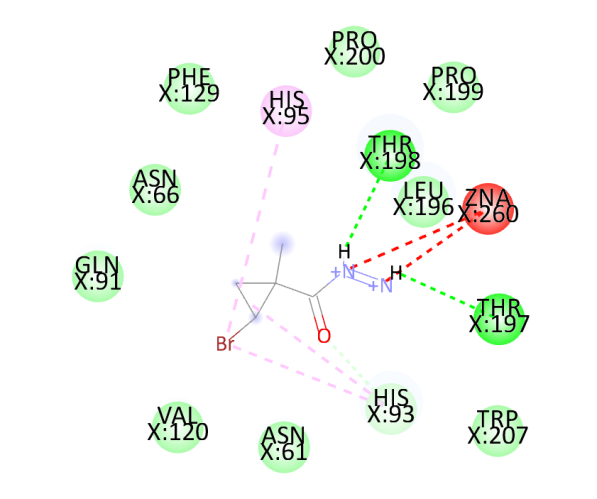** |
| **S54** | **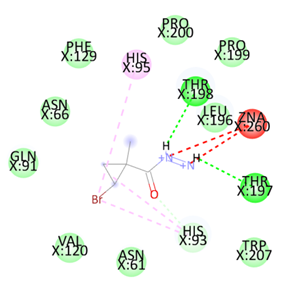** | **S55** | **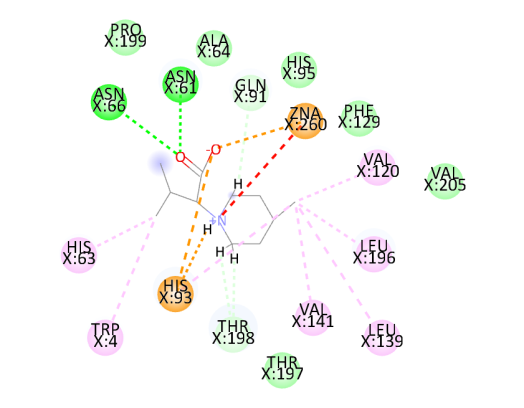** |
| **S56** | **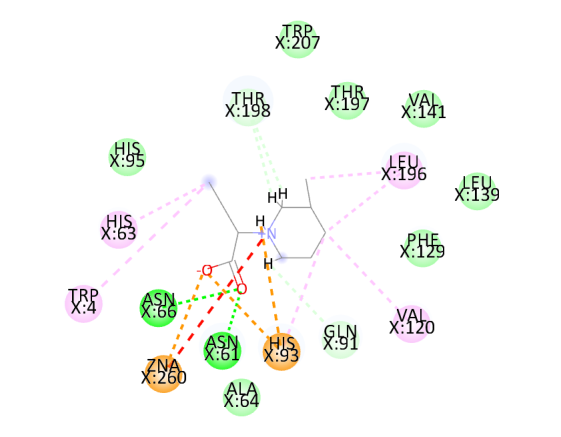** | **S57** | **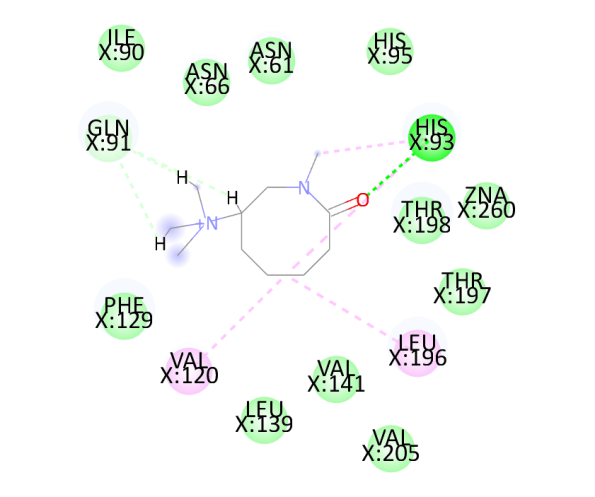** |
| **S58** | **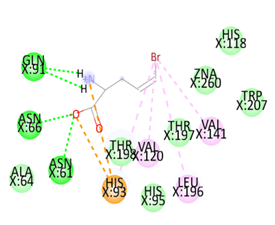** | **S59** | **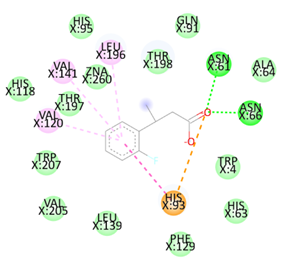** |
| **S60** | **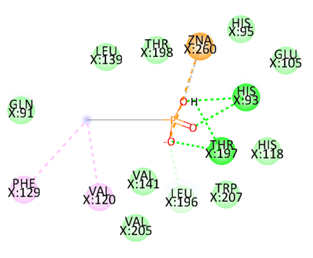** | **S61** | **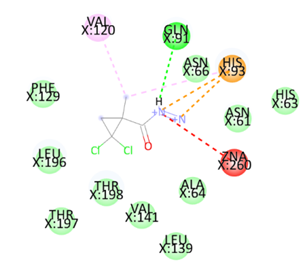** |
| **S62** | **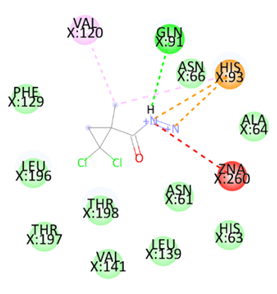** | **S63** | **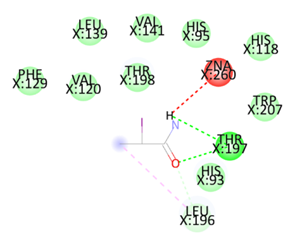** |
| **S64** | **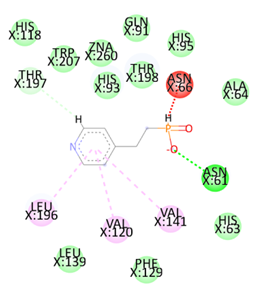** | **S65** | **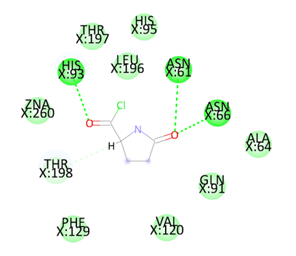** |
| **S66** | **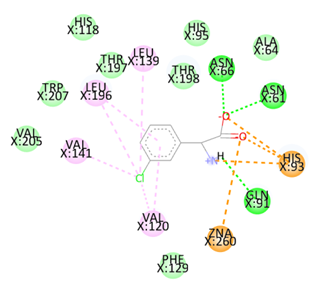** | **S67** | **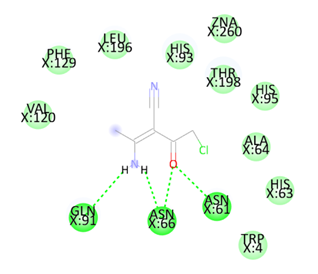** |
| **S68** | **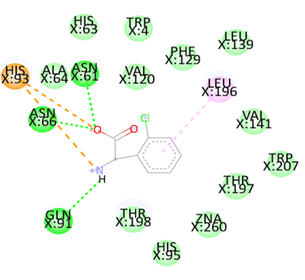** | **S69** | **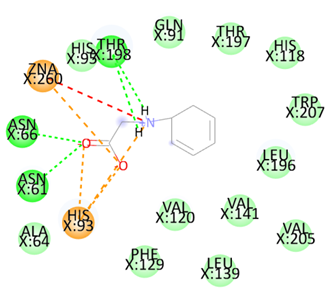** |
| **S70** | **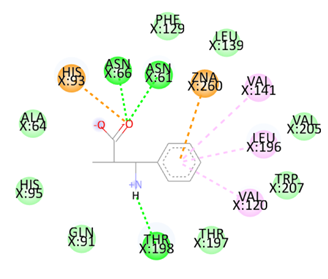** | **S71** | **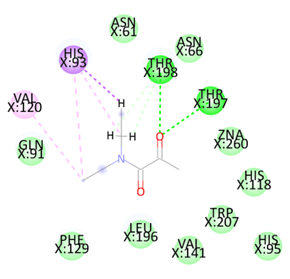** |
| **S72** | **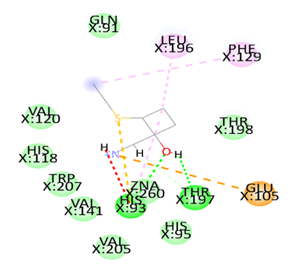** | **S73** | **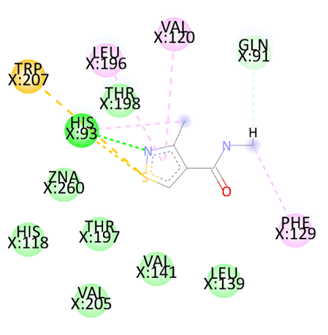** |
| **S74** | **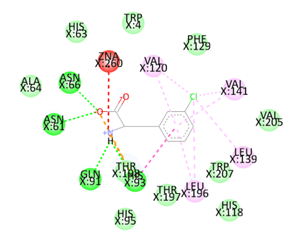** | **S75** | **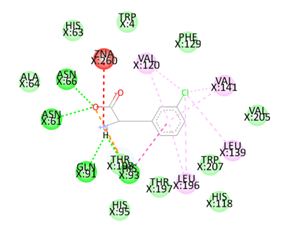** |
| **S76** | **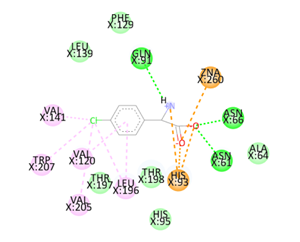** | **S77** | **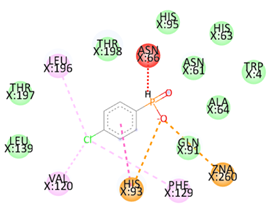** |
| **S78** | **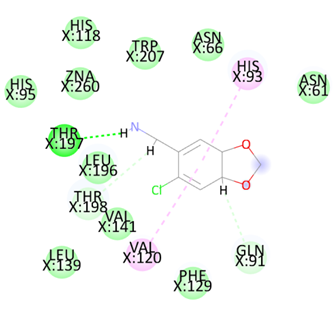** | **S79** | **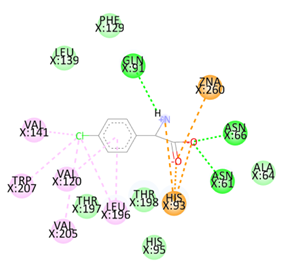** |
| **S80** | **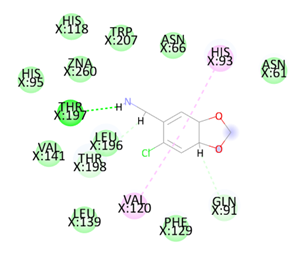** | **S81** | **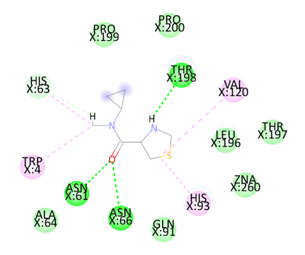** |
| **S82** | **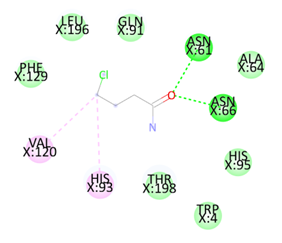** | **S83** | **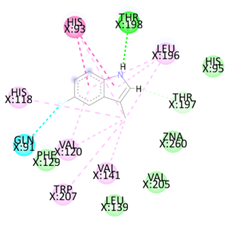** |
| **S84** | **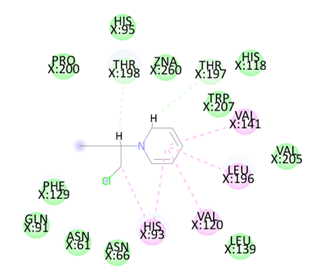** | **S85** | **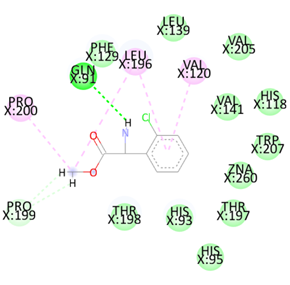** |
| **S86** | **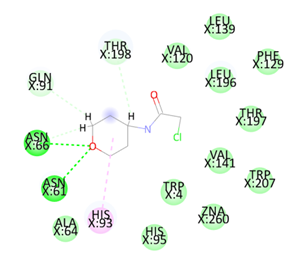** | **S87** | **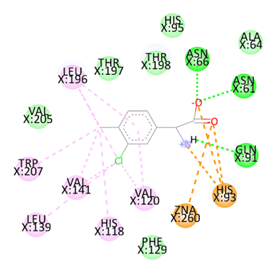** |
| **S88** | **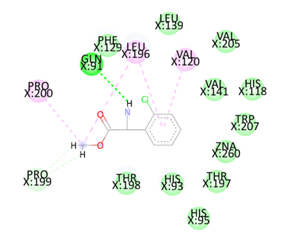** | **S89** | **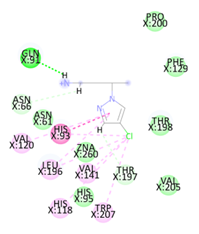** |
| **S90** | **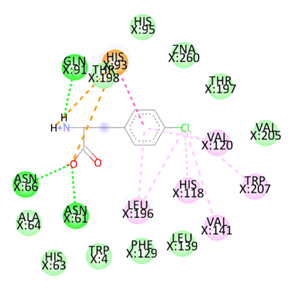** | **S91** | **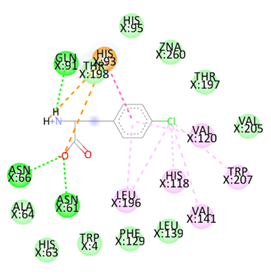** |
| **S92** | **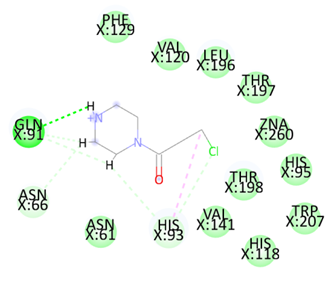** | **S93** | **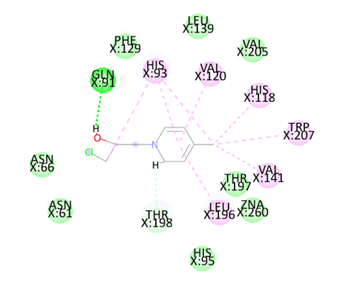** |
| **S94** | **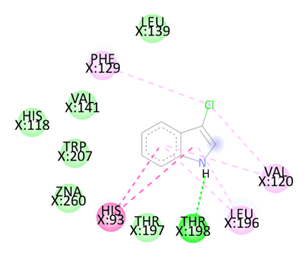** | **S95** | **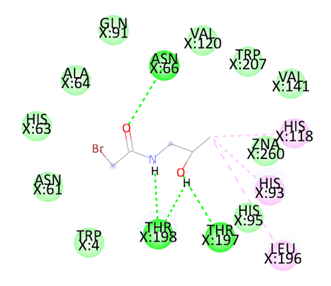** |
| **S96** | **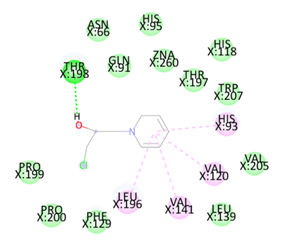** | **S97** | **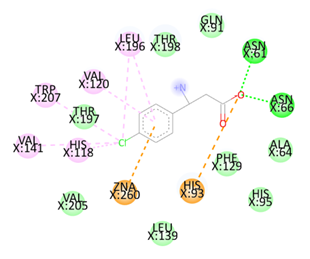** |
| **S98** | **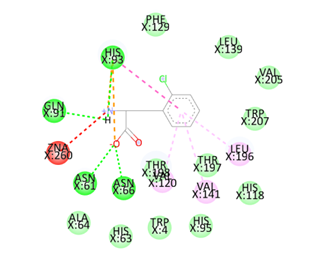** | **S99** | **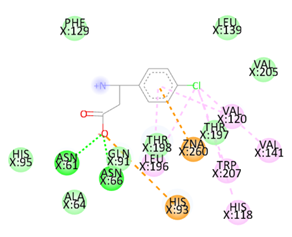** |
| **S100** | **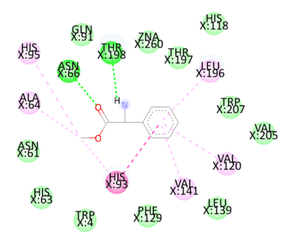** | **S101** | **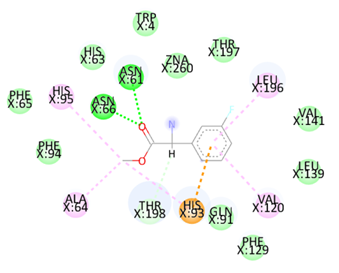** |
| **S102** | **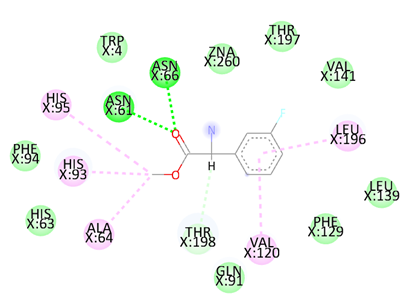** | **S103** |  |
| **S104** |  | **S105** |  |
| **S106** |  | **S107** |  |
| **S108** |  | **S109** |  |
| **S110** |  | **S111** |  |
| **S112** |  | **S113** |  |
| **S114** |  | **S115** |  |
| **S116** |  | **S117** |  |
| **S118** |  | **S119** |  |
| **S120** |  | **S121** |  |
| **S122** |  | **S123** |  |
| **S124** |  | **S125** |  |
| **S126** |  | **S127** |  |
| **S128** |  | **S129** |  |
| **S130** |  | **S131** |  |
| **S132** |  | **S133** |  |
| **S134** |  | **S135** |  |
| **S136** |  | **S137** |  |
| **S138** |  | **S139** |  |
| **S140** |  | **S141** |  |
| **S142** |  | **S143** |  |
| **S144** |  | **S145** |  |
| **S146** |  | **S147** |  |
| **S148** |  | **S149** |  |
| **S150** |  | **S151** |  |
| **S152** |  | **S153** |  |
| **S154** |  | **S155** |  |
| **S156** |  | **S157** |  |
| **S158** |  | **S159** |  |
| **S160** |  | **S161** |  |
| **S162** |  | **S163** |  |
| **S164** |  | **S165** |  |
| **S166** |  | **S167** |  |
| **S168** |  | **S169** |  |
| **S170** |  | **S171** |  |
| **S172** |  | **S173** |  |
| **S174** |  | **S175** |  |
| **S176** |  | **S177** |  |
| **S178** |  | **S179** |  |
| **S180** |  | **S181** |  |
| **S182** |  | **S183** |  |
| **S184** |  | **S185** |  |
| **S186** |  |  | |
